# MicroRNAs Form Triplexes with Double Stranded DNA at Sequence-Specific Binding Sites; a Eukaryotic Mechanism via which microRNAs Could Directly Alter Gene Expression

**DOI:** 10.1371/journal.pcbi.1004744

**Published:** 2016-02-04

**Authors:** Steven W. Paugh, David R. Coss, Ju Bao, Lucas T. Laudermilk, Christy R. Grace, Antonio M. Ferreira, M. Brett Waddell, Granger Ridout, Deanna Naeve, Michael Leuze, Philip F. LoCascio, John C. Panetta, Mark R. Wilkinson, Ching-Hon Pui, Clayton W. Naeve, Edward C. Uberbacher, Erik J. Bonten, William E. Evans

**Affiliations:** 1 Hematological Malignancies Program, St. Jude Children’s Research Hospital, Memphis, Tennessee, United States of America; 2 Department of Pharmaceutical Sciences, St. Jude Children’s Research Hospital, Memphis, Tennessee, United States of America; 3 High Performance Computing Facility, St. Jude Children’s Research Hospital, Memphis, Tennessee, United States of America; 4 Department of Structural Biology, St. Jude Children’s Research Hospital, Memphis, Tennessee, United States of America; 5 Molecular Interaction Analysis Laboratory, St. Jude Children’s Research Hospital, Memphis, Tennessee, United States of America; 6 Functional Genomics Laboratory, Hartwell Center for Bioinformatics & Biotechnology, St. Jude Children’s Research Hospital, Memphis, Tennessee, United States of America; 7 Computer Science and Mathematics Division, Oak Ridge National Laboratory, Oak Ridge, Tennessee, United States of America; 8 Doctoral Training Centre, University of Oxford, Oxford, United Kingdom; 9 Department of Oncology, St. Jude Children’s Research Hospital, Memphis, Tennessee, United States of America; 10 Department of Information Sciences, St. Jude Children’s Research Hospital, Memphis, Tennessee, United States of America; Ottawa University, CANADA

## Abstract

MicroRNAs are important regulators of gene expression, acting primarily by binding to sequence-specific locations on already transcribed messenger RNAs (mRNA) and typically down-regulating their stability or translation. Recent studies indicate that microRNAs may also play a role in up-regulating mRNA transcription levels, although a definitive mechanism has not been established. Double-helical DNA is capable of forming triple-helical structures through Hoogsteen and reverse Hoogsteen interactions in the major groove of the duplex, and we show physical evidence (i.e., NMR, FRET, SPR) that purine or pyrimidine-rich microRNAs of appropriate length and sequence form triple-helical structures with purine-rich sequences of duplex DNA, and identify microRNA sequences that favor triplex formation. We developed an algorithm (Trident) to search genome-wide for potential triplex-forming sites and show that several mammalian and non-mammalian genomes are enriched for strong microRNA triplex binding sites. We show that those genes containing sequences favoring microRNA triplex formation are markedly enriched (3.3 fold, p<2.2 × 10^−16^) for genes whose expression is positively correlated with expression of microRNAs targeting triplex binding sequences. This work has thus revealed a new mechanism by which microRNAs could interact with gene promoter regions to modify gene transcription.

## Author Summary

We provide physical evidence, using NMR, FRET and SPR, that purine or pyrimidine-rich microRNAs can form triplexes with complementary purine-rich sequences of duplex DNA and provide an algorithm (Trident) to search genome-wide for potential microRNA double-stranded DNA triplex-forming sites. Using this algorithm we document enrichment of microRNA triplex binding sites in mammalian and non-mammalian genomes. We found in primary leukemia cells from patients a significant over-representation of positively correlated microRNA and mRNA expression for genes containing sequences favoring microRNA-duplex DNA triplex formation, suggesting this as a mechanism by which microRNA may enhance gene transcription.

## Introduction

MicroRNAs influence a broad spectrum of biological processes and have been extensively characterized as negative regulators of gene function. By pairing with complementary sequences in messenger RNA (mRNA), they are known to down-regulate gene function by enhancing transcript degradation or sequestration, or via suppression of translation. MicroRNAs have also been shown to up-regulate mRNA transcript levels for some genes, but the mechanism(s) for increasing gene expression have not been fully elucidated [1–5]. One indirect mechanism by which microRNAs may up-regulate gene expression is via suppression of mRNAs encoding transcriptional suppressors. In addition, there are reports that interactions between microRNAs and gene promoter regions may play a more direct role in regulating the efficiency of gene transcription [1–3,6], for example by mediating *de novo* CpG methylation [7]. However, it is possible that there are other unidentified or not fully elucidated mechanisms by which microRNAs directly interact with genes to enhance gene transcription. Because double stranded DNA is capable of forming triple-helical structures through interactions with DNA or RNA in the major groove of the DNA duplex, we and others have postulated that microRNA may form triplex structures with duplex DNA via either Hoogsteen or reverse Hoogsteen hydrogen bonds, and thereby directly interacting with target DNA sequences in regulatory regions and gene promoters in the human genome, with the potential to alter gene function [8–10].

Here we provide direct physical evidence that microRNAs of sufficient length and sequence can bind to double stranded DNA to form hetero-triplex structures at specific target sequences in DNA. We computationally show that the human genome, as well as the genomes of multiple other species, contain DNA sequences with properties favoring microRNA triplex formation. We also show that those genes containing sequences favoring microRNA triplex formation are enriched (3.3 fold) for genes whose expression is positively correlated with expression of microRNAs targeting triplex binding sequences, indicating this as a potential mechanism via which microRNA can directly enhance gene expression.

## Results

### MicroRNA binding sites are enriched in multiple genomes

**Fig 1:**
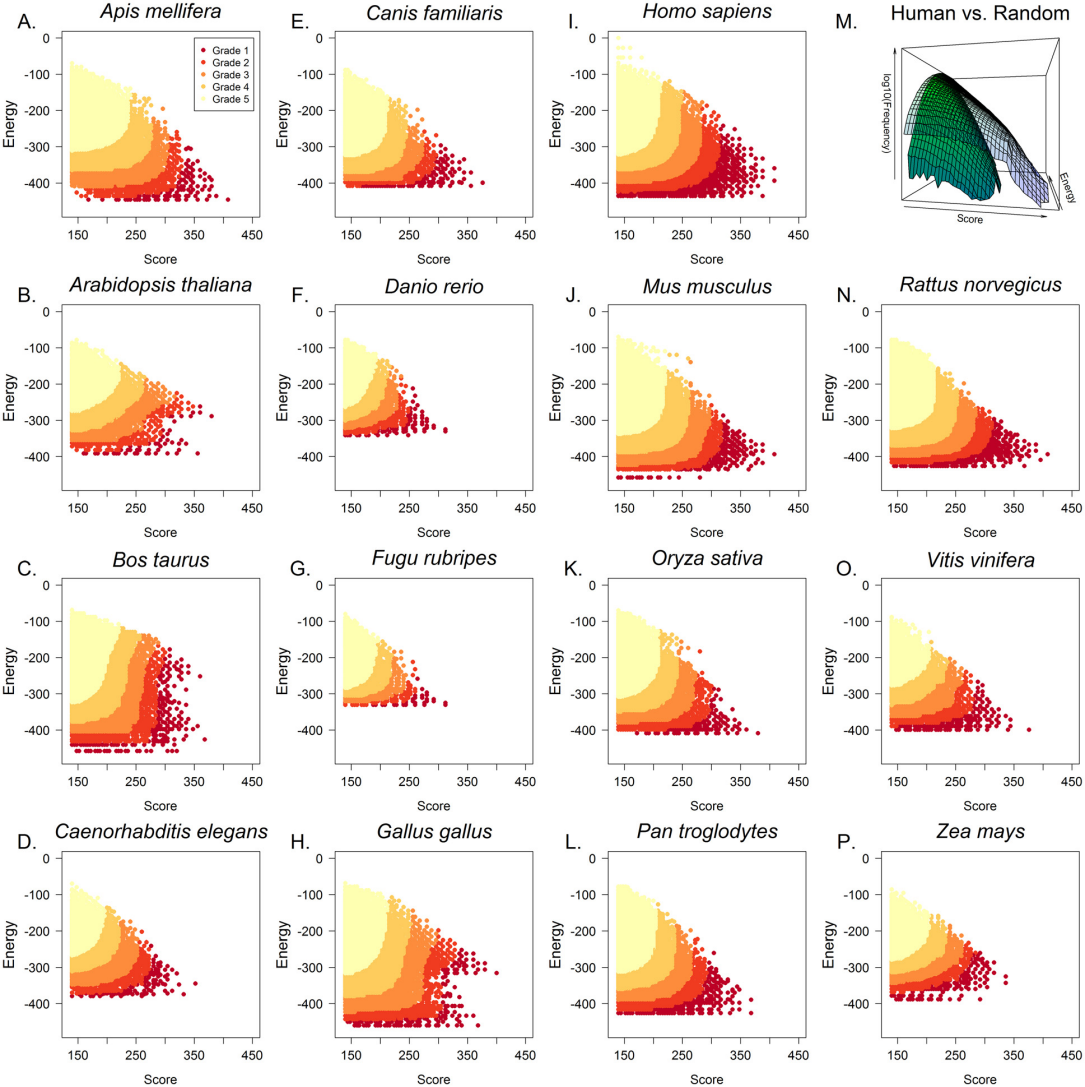
Triplex target sites are enriched in mammalian and non-mammalian genomes. **(A-P)** Genome-wide analyses of potential microRNA binding sites in genomic DNA were performed across fifteen species. The heuristic score (”Score”, x-axis) represents Hoogsteen or Reverse Hoogsteen base pair complementarity and Thermodynamic Energy (”Energy”, y-axis) represents the binding energy of the triplex (see Methods). Binding sites were categorized based on the number of hits with better score and energy. Grade 1 hits represent the 99.999^th^ percentile of triplex forming interactions, which are sequences most likely to participate in DNA-microRNA triplex formation. Subsequent grades are 10 fold lower in their percentile ranking (e.g. 99.99, 99.9, 99th percentiles). Additionally, randomly generated DNA sequences were analyzed against human microRNAs. The random DNA sequences (M, green surface) showed many orders of magnitude fewer binding sites than the human genome (M, blue surface) and the identified binding sites were of low quality (low score, high energy).

To assess the landscape of potential microRNA triplex binding sites in genomic DNA, we developed and implemented a computational algorithm (‘Trident’; http://trident.stjude.org) to identify Hoogsteen and reverse Hoogsteen interactions between single stranded oligonucleotides (i.e. microRNAs) and double-stranded oligonucleotides (i.e. duplex DNA). The algorithm identifies Hoogsteen and Reverse Hoogsteen interactions, independently searching for triplex forming units (e.g. Hoogsteen TA:U and CG:C; Reverse Hoogsteen TA:A and CG:G, in the form XY:Z, where Z represents the microRNA nucleotide) between stretches of polypurine genomic DNA and either polypurine or polypyrimidine third strand microRNAs, on an individual base level. For each detected triplex binding site (those sites capable of forming an interaction of multiple units), a thermodynamic binding energy and heuristic score was determined, with higher heuristic score and lower thermodynamic energy indicating stronger interaction. To determine the thermodynamic energy for binding, first order free energy calculations were performed to determine the amount of binding energy of each type of interaction. Heuristic score was determined based on the number of triplex forming pairs found between the interacting microRNA and double stranded DNA. Using this computational algorithm, we performed genome-wide binding site analyses on the genomes and microRNAs of several species, as well as randomly generated DNA sequences (Fig 1).

For each genome analyzed, the genome-wide results with heuristic scores greater than or equal to 140 (≥7 triplex forming units) were ranked and categorized on the basis of the number of other identified binding sites with a better energy/score combination (having lower energy and higher score). Those interactions with an energy/score ranking greater than the top 0.001% were classified as grade 1 hits, with grades 2–5 being assigned to interactions with energy/score rankings with successive ten-fold lower criteria (e.g. top 0.01%, 0.1%, and 1%). This analysis revealed a highly significant enrichment of microRNA triplex binding sites in all genomes analyzed (Fig 1), including *Homo sapiens* (Fig 1I), *Mus musculus* (Fig 1J), *Rattus norvegicus* (Fig 1N), *Caenorhabditis elegans* (Fig 1D) and *Arabidopsis thaliana* (Fig 1B), when compared to random DNA sequences analyzed for microRNA binding sites. Comparing the log-transformed interpolated frequency of identified binding sites of human genome and randomly-generated DNA sequences, reveals a distinct enrichment (p-value <2.2 × 10^−16^) in low energy and high score hits (Fig 1M).

### Imbalance of purine:pyrimidine sequences are favored in microRNAs involved in identified triplex binding sites

**Fig 2:**
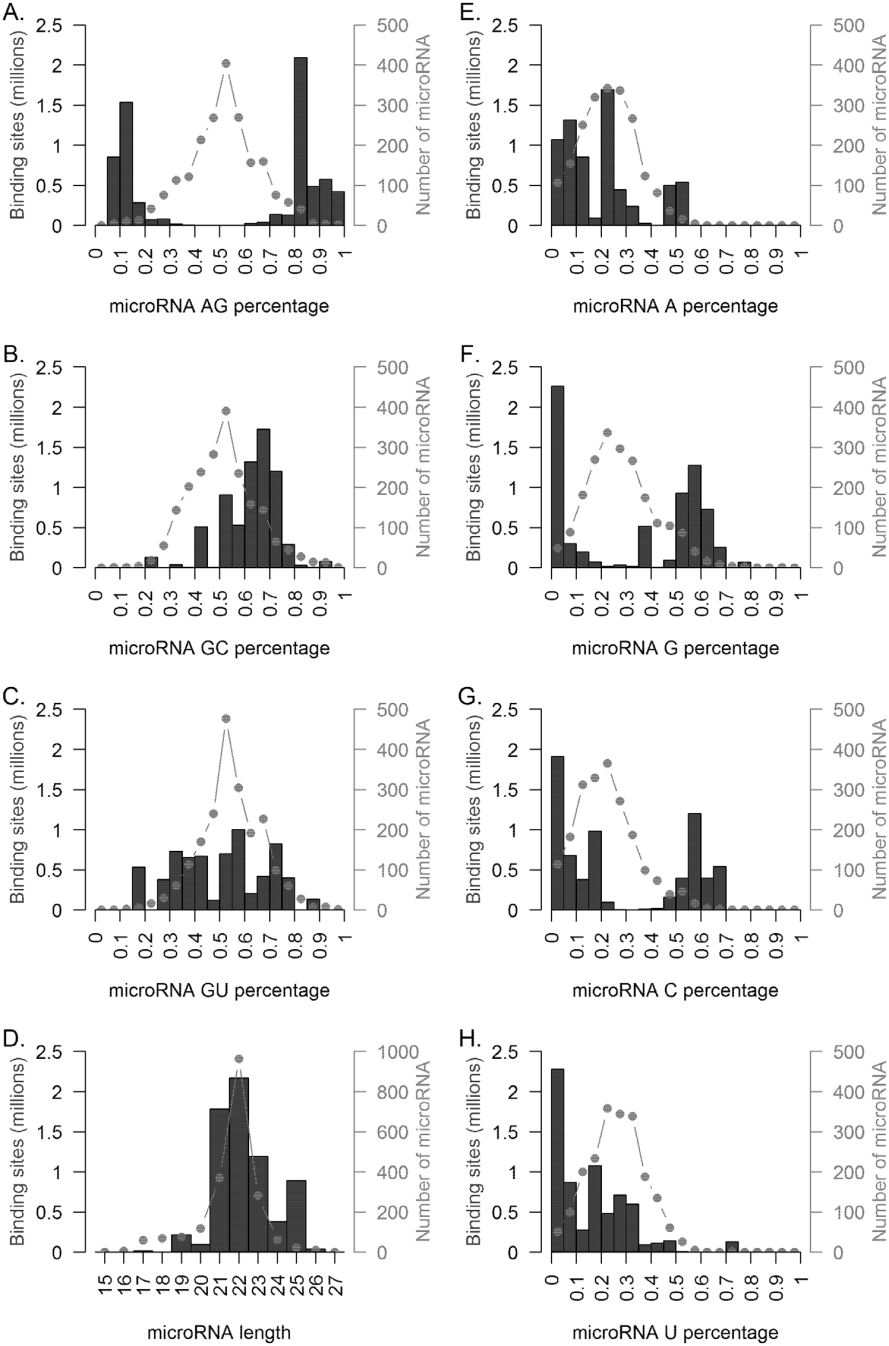
Characteristics of triplex forming microRNA. The top 1 percent of Homo sapiens triplex interactions (grades 1–4) were characterized by **(A-C)** microRNA dinucleotide frequency, **(D)** microRNA length, and **(E-H)** single nucleotide frequency, and compared to these same characteristics for all human microRNAs. The percentage of purine content was the largest discriminating factor in predicting triplex formation, with the majority of binding sites having greater than 75% purine or pyrimidine content (A). Higher GC content (B), length between 21 and 25 nucleotides (D), greater than or less than average G or C content (F and G), and lower than average U content (H) also predicted triplex formation.

To identify potential novel classes of microRNA that bind to double stranded DNA, we assessed the sequence content of the microRNAs computed to participate in triplex formation. Comparisons of the identified binding site frequencies and microRNA sequence content revealed a marked enrichment (Fig 2A) in identified binding sites for microRNAs exhibiting imbalanced purine to pyrimidine content (e.g. high purine content or high pyrimidine content). Notably, the distribution of purine content in known human microRNAs has a mean of approximately 50%, suggesting that those microRNAs with imbalanced purine to pyrimidine content were responsible for a disproportionate number of triplex binding interactions. Indeed, microRNAs with greater than 75% purine or greater than 75% pyrimidine content accounted for 95.3% of binding sites (Grades 1–4) identified and this distribution was significantly different than the distribution of purine content in known human microRNAs (2-sample test for equality of proportions with continuity correction p-value <2.2 × 10^−16^). This enrichment was significantly different as compared to imbalanced GC (Fig 2B) where only 7.8% of identified binding sites had microRNAs with greater or less than 25% GC content (2-sample test for equality of proportions with continuity correction p-value <2.2 × 10^−16^) or imbalanced microRNA GU (Fig 2C) content where only 15.8% of hits had microRNAs with greater or less than 25% GU content (2-sample test for equality of proportions with continuity correction p-value <2.2 × 10^−16^). In addition to purine:pyrimidine content, imbalance being an important determinant of triplex formation, lower than average U content (Fig 2H), higher than average G (Fig 2F) or C (Fig 2G) content also predicted affinity for double stranded DNA binding. We found no evidence that average A content (Fig 2E) was a determinant of microRNA binding to double stranded DNA.

### MicroRNAs bind double stranded DNA

**Fig 3:**
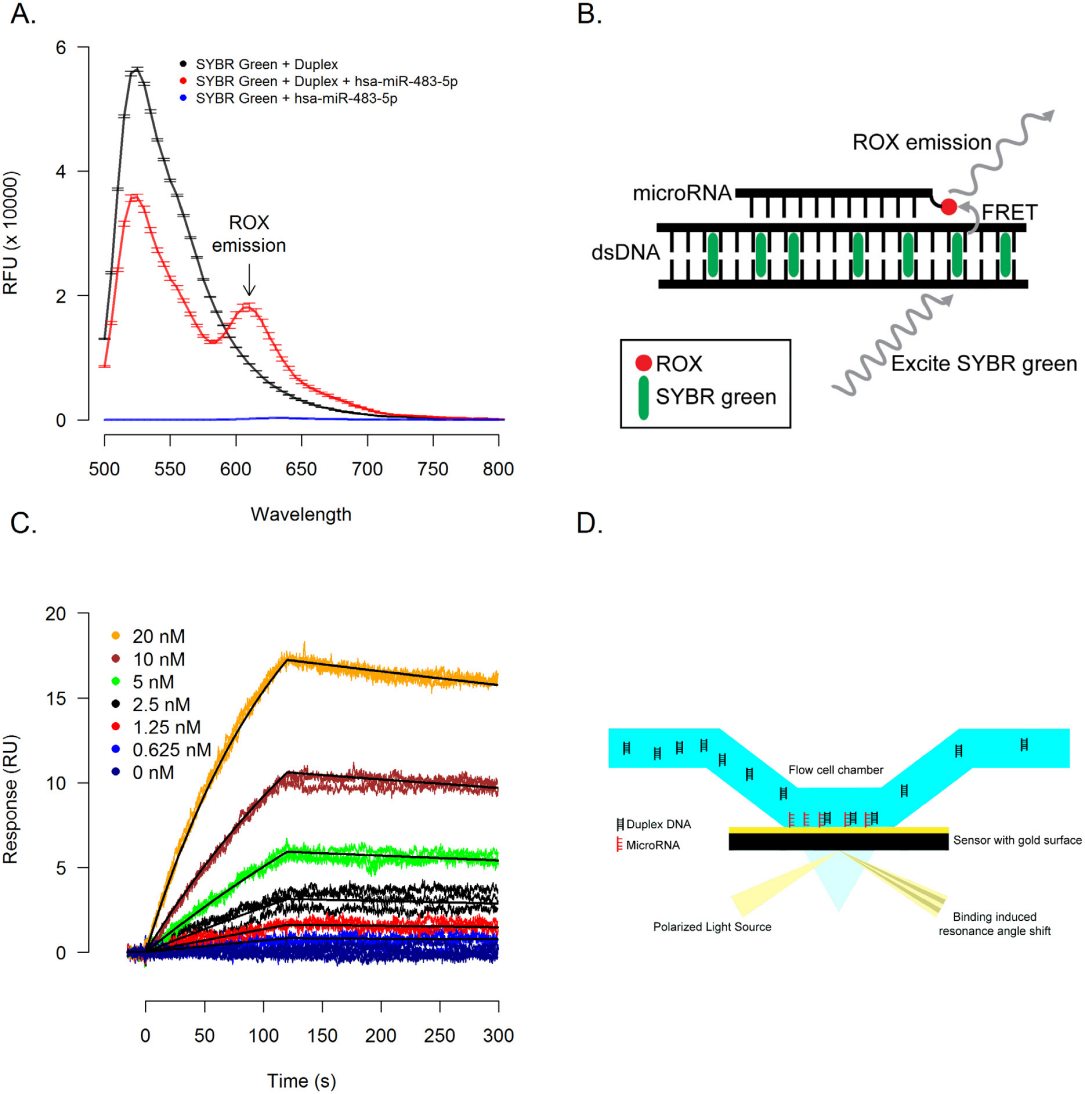
MicroRNAs form triplex structures with DNA. **(A)** Duplex DNA identified by genome-wide screens of binding sites was incubated in presence or absence of a synthesized hsa-miR-483-5p with a 3’ ROX label to perform a FRET assay to detect triplex formation (illustrated in **3B**). In the absence of ROX labeled hsa-miR-483-5p (**3A**, black line) a single emission peak at 520nm is observed which, with the addition of ROX labeled hsa-miR-483-5p (**3A**, red line), is diminished and a second FRET induced emission peak at 610nm is observed. **(C)** In a complementary surface plasmon resonance (SPR) based assay (illustrated in **3D**), a 3’ biotin labeled hsa-miR-483-5p was immobilized and duplex DNA was introduced in triplicate in a 2-fold dilution series starting at 20 nM.

To verify that microRNAs are capable of physical interaction and binding to double-stranded DNA, we designed orthogonal methods to directly interrogate binding. A fluorescence resonance energy transfer (FRET) based method (Fig 3B) to detect triplex formation was designed such that a double stranded DNA intercalating dye (SYBR Green II), when excited at 480 nm, transfers energy to a carboxy-X-rhodamine (ROX) molecule covalently coupled to a triplex forming RNA. Decreased emission at 520 nm of the double-stranded DNA intercalating donor dye corresponds with increased emission of ROX acceptor dye at 610 nm. Utilizing this method, we detected the interaction of hsa-miR-483-5p (a microRNA high in purine content) and a double stranded DNA (an identified Hoogsteen binding site in our genome wide screen), as evidenced by the decreased SYBR Green emission and increased ROX emission (Fig 3A).

Utilizing a complementary surface plasmon resonance (SPR) based method, we verified that hsa-miR-483-5p immobilized via biotin based coupling to the detector surface (Fig 3D) was able to bind duplex DNA, but neither hsa-miR-1 nor hsa-miR-98 (microRNAs with mixed purine/pyrimdine content) was able to bind complementary double stranded DNA (S1 Fig). Kinetic analysis (Fig 3C) yielded an association rate constant (*k*_a_) of 3.96 (± 0.01) × 10^5^ M^-1^s^-1^, a dissociation rate constant (*k*_d_) of 5.01 (± 0.07) × 10^−4^ s^-1^ and an equilibrium dissociation constant (*K*_D_) of 1.27(± 0.02) nM.

**Fig 4:**
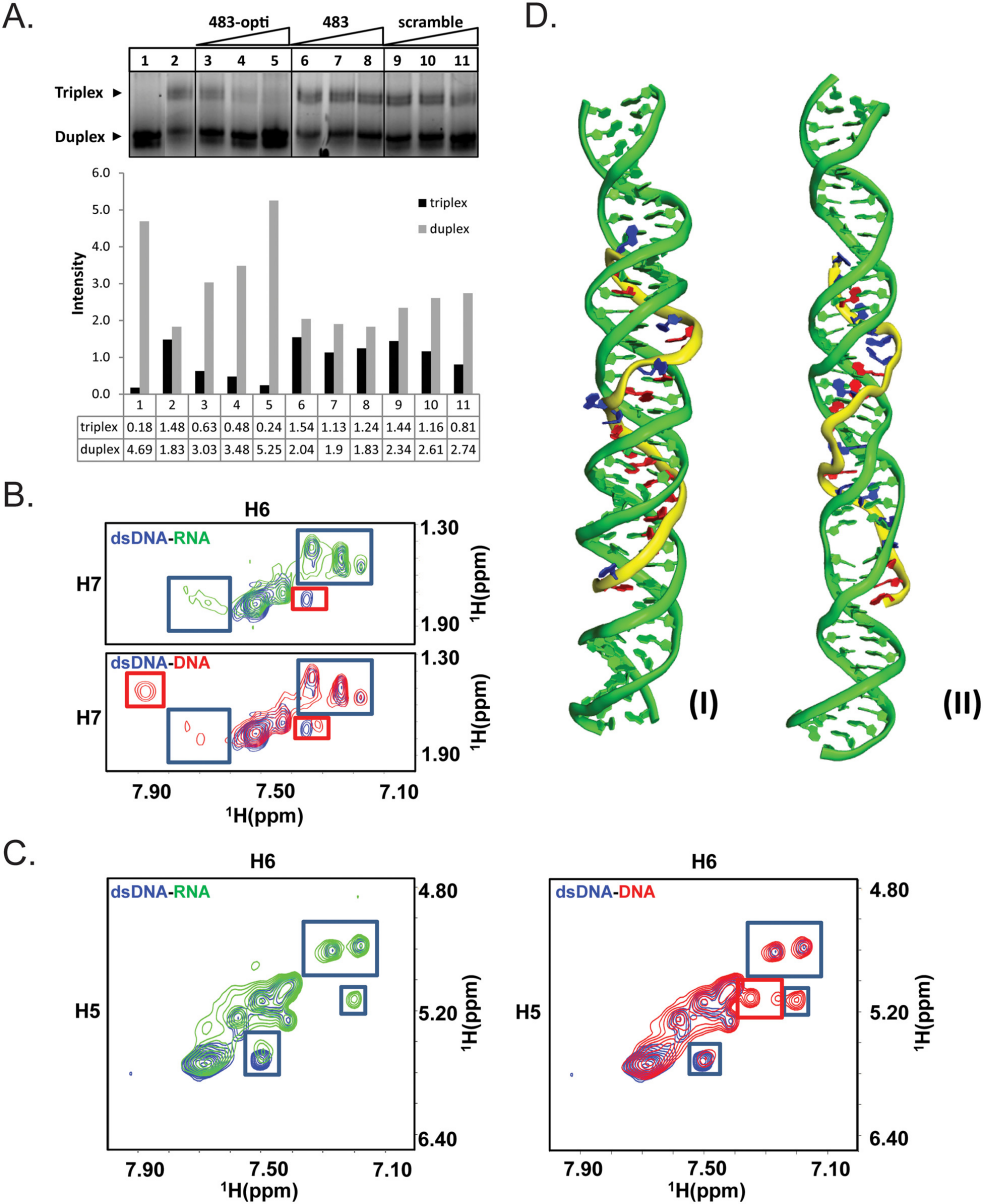
Detection of DNA-DNA and RNA-DNA triplexes by EMSA and NMR, and molecular modeling of miRNA-duplex DNA triplex. **(A)** EMSA; 5’ ROX-labeled hairpin duplex DNA (0.1 μM) was incubated for 3-hrs at 22°C in the presence (lanes 2–11) or absence (lane 1) of 2.5 μM 483-opti DNA oligo, and increasing concentration (30, 60, 150 μM) of Hoogsteen bond-optimized hsa-miR-483-5p (483-opti, lanes 3–5), hsa-miR-483-5 (483, lanes 6–8), or a scrambled RNA oligo (Scramble, lanes 9–11). Duplexes and triplexes were resolved on a 20% non-denaturing acrylamide gel, and the ROX-signal visualized. Triplex of 483-opti DNA oligo and duplex DNA is readily detected (lane 2). The 483-opti RNA oligo competes with 483-opti DNA oligo for binding to duplex DNA which is evident by increased amounts of duplex DNA and decreased amounts of triplex (compare lanes 3–5 with lane 2). Hsa-miR-483-5p (483) and scrambled RNA, because of the fewer number of favorable Hoogsteen bonds, did not compete with the 483-opti DNA oligo for binding to duplex DNA (lanes 6–7 and 9–10, respectively). **(B-C)** NMR; Two-Dimensional (2D) [^1^H, ^1^H] TOCSY spectra of free single stranded hairpin duplex DNA (blue contours), hairpin duplex DNA combined with hsa-miR-483-5p RNA oligo (green contours; 1:1.5 ratio), and hairpin duplex DNA with single stranded DNA oligo with the same sequence as hsa-miR-483-5p (red contours; 1:1ratio). **(B)** Thymidine cross-peaks between H6 and H7 (methyl), and **(C)** cytosine cross-peaks between H5 and H6. Single stranded RNA (hsa-miR-483-5p) or single stranded DNA with hairpin duplex DNA show similar improvement in peak the intensities, and similar chemical shift perturbations/appearance of new peaks highlighted in blue boxes, suggesting that single stranded DNA and single stranded RNA of the same sequence bind to DNA duplex in a similar manner; the major differences (peaks in red boxes) are one peak among thymidine cross-peaks, showing an intermediate change (peak disappearing) with singe stranded RNA while saturated with hairpin duplex DNA, and two new peaks among cytosine cross-peaks showing much higher intensities with single stranded DNA, indicating that the latter DNA binds to duplex DNA duplex with higher binding affinity than RNA, consistent with the results obtained by EMSA. **(D)** Molecular model of hsa-miR-483-5p-DNA triplex. (I): the model of predicted miRNA and corresponding DNA duplex sequences (16 favorable Hoogsteen pairings). All predicted Hoogsteen base pairs are well maintained after removal of positional and distance restraints(II): negative control (antisense hsa-miR-483-5p) of model with 9 favorable Hoogsteen pairings. Both RNA and DNA duplex are largely twisted and nearly all predicted Hoogsteen pairings cannot be stably maintained. Residues in favor of Hoogsteen hydrogen bond formation are shown in red while the others are shown in blue.

To corroborate our findings by FRET and SPR (Fig 3), we also performed EMSA experiments to document binding between hsa-miR-483-5p and ROX-labeled 24-bp hairpin duplex DNA, but reasoned that the interaction between duplex DNA and microRNA is likely transient and not readily detectable by EMSA, whereas single stranded purine-rich DNA oligonucleotides were more likely to form stable triplexes that are detectable by EMSA [11]. To test this theory, we performed EMSA experiments which documented that Hoogsteen bond-optimized hsa-miR-483-5p RNA (483-opti) competed with 483-opti DNA oligo with the same nucleotide sequence for binding to duplex DNA, resulting in decreased amounts of triplex DNA and increased amounts of duplex DNA (Fig 4A, lanes 3–5), providing evidence that Hoogsteen bond-optimized hsa-miR-483-5p (483-opti) binds to duplex DNA. In contrast, because of fewer favorable Hoogsteen bonds, hsa-miR-483-5p (Fig 4A, lanes 6–8) and an RNA oligo with scrambled sequence (Fig 4A, lanes 9–11) did not compete with the DNA oligo for binding to duplex DNA. In addition, EMSA experiments with 11-nucleotide DNA and RNA oligos corresponding to the 5’ and 3’ regions of Hoogsteen bond-optimized hsa-miR-483-5p, did not result in detectable triplex formation with the hairpin duplex DNA (S2A Fig), suggesting that sequence, purine content, and length of the microRNA are important factors influencing binding of microRNA to duplex DNA. These competition EMSA results indicate that microRNA-duplex DNA triplex formation is transient, and better suited for detection by more sensitive methods such as FRET, SPR, and NMR.

### Structural confirmation of triplexes via NMR

To corroborate our findings by EMSA, we performed Two-Dimensional (2D) [^1^H, ^1^H] NMR of 24 bp hairpin duplex DNA in presence and absence of 22 nucleotide single stranded hsa-miR-483-5p RNA oligo and DNA oligo with the same sequence (Fig 4B and 4C). Overall single stranded RNA (hsa-miR-483-5p) and single stranded DNA mixtures with hairpin duplex DNA show similar binding profile, with similar improvement in the peak intensities and chemical shift perturbations with the appearance of new peaks highlighted in blue boxes (Fig 4B and 4C), suggesting that single stranded DNA and single stranded RNA of the same sequence bind to DNA duplex in a similar manner; the major differences (in red boxes) are one peak among thymidine cross-peaks (Fig 4B), showing an intermediate change (peak disappearing) with single stranded RNA while saturated with hairpin duplex DNA, and one additional peak probably coming from the loop region; two new peaks among cytosine cross-peaks (Fig 4C) showing much higher intensities with single stranded DNA, indicating that single stranded DNA binds to duplex DNA duplex with higher binding affinity than RNA, consistent with the results obtained by EMSA.

We modeled DNA-microRNA triplex of double stranded DNA and hsa-miR-483-5p *in silico* by simulated annealing with distance restraints derived from Hoogsteen base pairing, and subsequently simulated the structure by Langevin molecular dynamics in generalized born solvent model. (Fig 4D). The model shows that the hsa-miR-483-5p microRNA strand is binding to the targeted DNA duplex region in an antiparallel mode, and most of the predicted Hoogsteen hydrogen bonds are reasonably well maintained even after the removal of external restraints in the top rated predicted sequence (Fig 4D-I). By comparison, the negative control model of triplex with the reverse RNA sequence cannot maintain Hoogsteen pairs during MD simulation (Fig 4D-II). This binding model is overall consistent with the chemical shift changes observed for the cytosine and thymidine signals of DNA duplex upon single stranded RNA binding (Fig 4B and 4C). Overall, the molecular modeling is consistent with EMSA and NMR results, which suggests that longer purine-rich RNA can form triplex with DNA duplex in an antiparallel manner. In contrast, there was complete overlap between the two TOCSY spectra of free double stranded DNA and that of double stranded DNA combined with a shorter, 11-nucleotide truncated hsa-miR-483-5p oligo (S2B and S2C Fig), indicating that this short RNA was incapable of triplex formation, which is consistent with a previous report that shorter purine-rich RNA cannot form triplex with double stranded DNA[11]. This confirms our EMSA results that, besides the sequence and purine-content, the length of the microRNA is an important factor influencing triplex formation between double stranded DNA and microRNA.

### MicroRNAs that form triplexes with duplex DNA are more frequently positively correlated with gene transcripts

**Fig 5:**
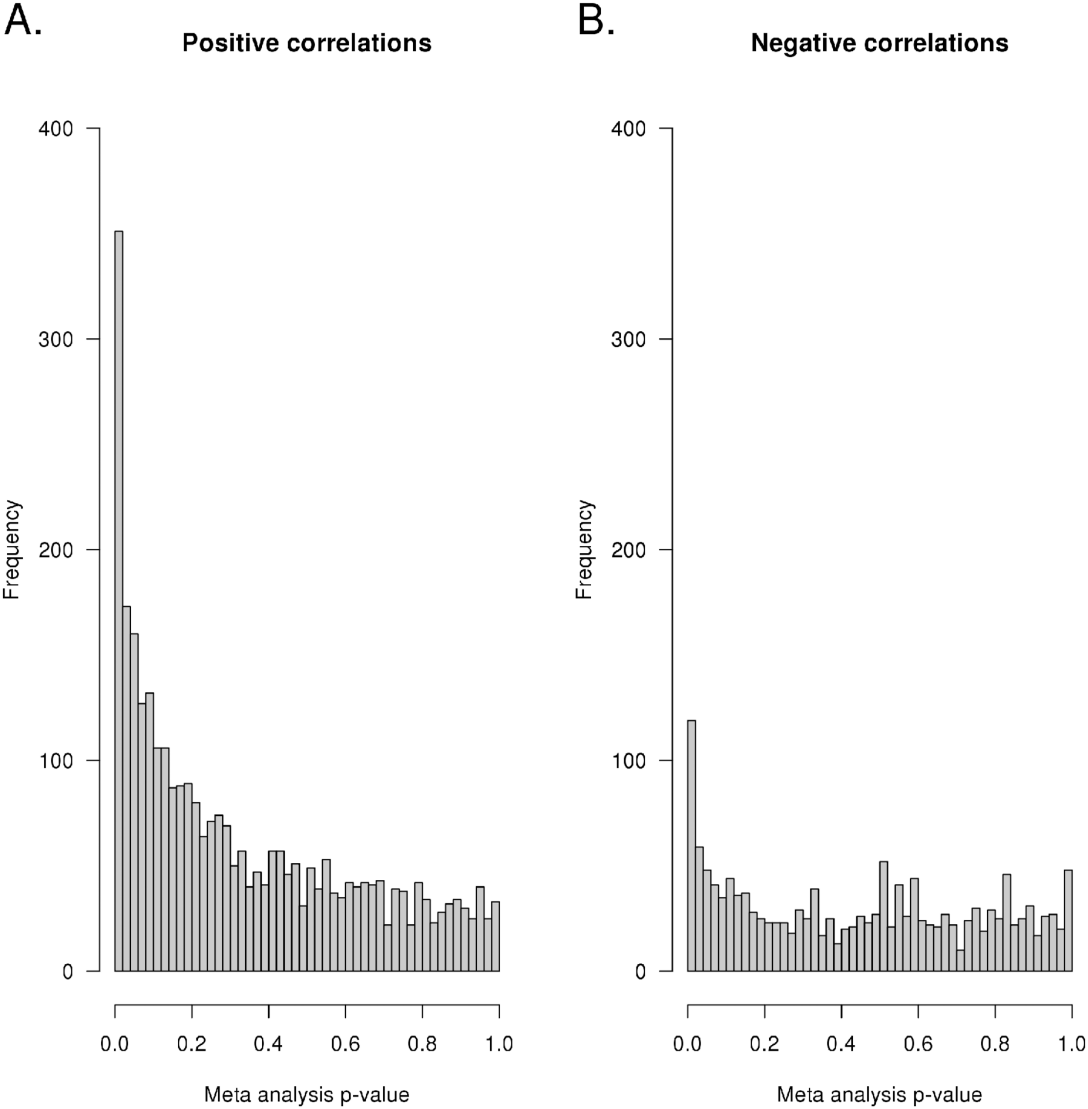
Higher expression of microRNAs forming triplex structures with duplex DNA is more frequently associated with increased gene expression. MicroRNA and mRNA expression were measured in leukemia cells (ALL) obtained at the time of diagnosis from two cohorts of patients (St. Jude Protocols Total 15 and Total 16). Genome-wide linear correlations between microRNA expression and mRNA expression calculated to form grade 1 triplex structures were assessed in each cohort separately and then a meta-analysis was performed. **(A)** The distributions of Spearman p-values for associations with positive or **(B)** negative correlations are shown. Over-representation of small p-values for positive associations was significantly enriched as compared to negative associations.

To further interrogate our genome-wide assessments of microRNA binding sites, we measured microRNA and mRNA expression levels in primary leukemia cells isolated from two independent cohorts of patients enrolled on either St. Jude Total Study 15 or Study 16 protocol for children with newly diagnosed acute lymphoblastic leukemia (ALL) and assessed their correlations. Spearman correlation analysis was performed in each cohort separately, cross comparing every microRNA to every mRNA probe set. Meta-analysis combining the results from both cohorts revealed that for those microRNA-gene pairings with an identified grade 1 Trident binding site (within 5000 base pairs up and down stream of the gene), there was a marked enrichment of significant positive correlations. There were 2639 genes that contained a duplex DNA sequence (within 5000bp of the gene) estimated via Trident to have a grade 1 interaction with a microRNA by either Hoogsteen or reverse Hoogsteen interaction. As shown in Fig 5, of these 2639 genes, there was a highly significant enrichment (3.3-fold, p<2.2 × 10^−16^) for positively correlated mRNA-microRNA pairs (n = 206 with p-values <0.01), compared to negatively correlated microRNA-mRNA pairs (n = 62 with p-values <0.01).

## Discussion

Here we provide multiple lines of direct physical evidence that microRNAs can bind to double stranded DNA to form triplex structures and show that mammalian and non-mammalian genomes are enriched with microRNA triplex binding sites. Regulation of gene expression by microRNA binding directly to messenger RNA is well established. However, several studies have suggested the existence of a mechanism of transcriptional activation by microRNA binding to double stranded DNA, but no definitive mechanism for this microRNA-DNA interaction has been elucidated [1–3,12]. Additionally, the presence of microRNA in the nucleus[13] and a molecular mechanism for mature microRNA import into the nucleus[14] underscores the potential for nuclear functions of microRNAs. Indeed, there have been reports of triplex structures involving RNA-RNA interactions[15], but microRNA-duplex DNA interactions warrant further study[16]. Purine bases have more than one face from which they can form hydrogen bonds, which allows them to simultaneously participate in Watson-Crick pairings and either Hoogsteen or Reverse Hoogsteen pairings. When a run of purines on one strand of the duplex occurs, a third strand of either DNA or RNA with the correct Hoogsteen complementarity can interact with the major groove of DNA to form a triple helix through the formation of Hoogsteen or Reverse Hoogsteen hydrogen bonds. Informatics approaches for identifying homopurine sequences in genomes have been reported previously [8–10,15,17,18], however these methods did not contextualize these sequences in terms of potential for triplex formation with known microRNA species. Previous studies focused on the identification and interrogation by EMSA of stable interactions between target duplex DNA and purine or pyrimidine rich single stranded DNA or relatively short (12–14 mer) RNA oligonucleotides, with mostly favorable Hoogsteen pairings[11,19]. Indeed, our studies (using either EMSA or NMR) showed that purine rich short microRNA (e.g., 11 nucleotides) do not form stable triplex structures, consistent with previous reports[11], whereas longer microRNA (e.g., 22 nucleotides) with the appropriate sequence form triplex structures with duplex DNA as documented by FRET, SPR and NMR. These RNA-duplex DNA triplexes were not sufficiently stable to withstand gel electrophoresis for detection by EMSA, a known limitation of EMSA[20], but microRNA with appropriate sequence displaced DNA molecules from DNA-DNA triplexes, as documented by EMSA. The development and refinement of more powerful experimental tools such as FRET, SPR, and NMR have made it possible to identify transient interactions that may occur commonly in cell nuclei, and we used each of these methods to document formation of microRNA-duplex DNA triplexes. Analogous to transient protein-protein and DNA/RNA-protein interactions, transient formation of microRNA-duplex DNA triplexes may have as much biological importance as more stable interactions (reviewed in [16]).

Interestingly, helicases capable of unwinding intramolecular DNA triplex structures are known [8,21] and it is conceivable that this triplex mediated unwinding is a mechanism by which microRNAs can mediate transcriptional activation. Mutations in the human ChlR1 gene, which encodes a triplex-preferring helicase, result in the genetic disorder Warsaw breakage syndrome, characterized by defects in genome maintenance. Cells that were depleted of ChlR1 had increased triplex DNA content and double-stranded breaks[22]. Triplex Structures may promote genome instability by stalling replication forks at (GAA)_n_ repeats and inhibiting replication of DNA. Friedreichs ataxia, the most common form of ataxia in humans, is caused by the expansion of a (GAA)_n_ repeat in intron 1 of the Frataxin gene, which in turn results in transcriptional silencing, presumably because of the triplex-forming potential of the (GAA)_n_ repeat[23]. This suggests that, not only may the formation of DNA triplexes be a well-conserved and essential mechanism to regulate gene transcription, but that stable or prolonged triplex formation may have undesirable consequences. Therefore, it is likely that organisms would have multiple mechanisms to destabilize DNA triplexes. Besides the expression of triplex-specific helicases and potentially other ways to disrupt triplexes, the relatively weak or transient binding of microRNAs to target sites in the genome may constitute another mechanism against DNA-DNA triplex formation. Indeed, our EMSA experiments document that microRNA can disrupt DNA-DNA triplexes in a sequence specific manner, and results from both EMSA and NMR indicate that microRNA-duplex DNA triplexes are relatively transient. The binding to duplex DNA by other microRNAs, and characterization of the effects on gene transcription and downstream phenotypic consequences, merit further study to determine the biological function of such microRNA-DNA interactions.

We have shown that DNA sequences that favor microRNA-DNA triplex formation exist throughout the genome of humans and numerous other species. While microRNA-mRNA binding site searches has been done previously [24], methods presented here represent a novel technique for assessing microRNA-DNA binding through Hoogsteen and reverse Hoogsteen interactions. The Trident algorithm resembles microRNA-mRNA binding site algorithms (e.g. miRanda) in its search of binding site pairs, however the algorithm has adapted in it rules for base pair binding. Trident binding rules assign a thermodynamically determined energy to C:G and U:A pairs or G:G and A:A triplex pairs when searching for Hoogsteen and Reverse Hoogsteen binding, respectively. Thermodynamic energies reported by Trident and miRanda differ as well. First order free energy calculations were performed on each possible base pair permutation in a constrained microRNA-DNA triplex and these pair-wise interaction energies are the sum for each base pair in the triplex. Additionally, Trident does not add weighting to base pairs in seed sites due to the symmetric nature of the duplex DNA, microRNA interaction.

In addition to the binding site search algorithm, Trident provides a toolkit for analyzing potential triplex structures. Binding site heuristic is developed using a post-processing sequence provided by Trident. For computational efficiency, the entire process was designed and run on a Hadoop cluster. However, each part of the sequence was built to be run as a standalone Python application as well. In addition to statistical analyses, tools are provided to visualize triplex search data, including an interactive web portal (http://trident.stjude.org). While similar websites may be found for microRNA-mRNA binding sites, Trident goes beyond search. Using JBrowse [25], users can interactively view genome, Trident and gene data, which are all tied to database records. Notably, we have validated the physical interaction of microRNA and duplex DNA using four separate physical methods of triplex detection (FRET, SPR, EMSA, and NMR) and used molecular dynamics to model the interaction. These physical interactions are buttressed by empirical measurement of microRNA and mRNA correlations in two separate cohorts of patients with ALL, revealing a marked enrichment (3.3 fold) for grade 1 Trident binding near genes whose expression is positively correlated with expression of microRNAs.

In conclusion, although intermolecular DNA triplex structures have been detected in cell nuclei, suggesting their possible involvement in gene regulation [26,27], our study provides direct physical evidence of heterotriplex formation involving microRNA and duplex DNA. Moreover, these triplexes involve microRNAs that are either purine or pyrimidine rich (>75%) and bind to specific targeted sequences in duplex DNA. We also show that microRNAs that are predicted to form sequence specific triplexes with duplex DNA are enriched for those that are positively correlated with mRNA transcript levels of the targeted genes (p<2.2 x 10^−16^). The molecular action of these heterotriplexes may include the inducement of conformational changes in the immediately surrounding DNA, including a slight unwinding [28], a potential mechanism for promoting transcription. Alternatively, triplex specific binding proteins could conceivably alter the topography of gene promoter regions such that transcription factors are able to bind [29]. Our findings provide a platform for discovery of new functions of microRNA in both disease and non-disease states.

## Methods

### Patient samples

Written informed consent was obtained from parents/guardians and assent from patients, as appropriate. The research and use of these samples were approved by the institutional review board at St. Jude Children’s Research Hospital.

### Gene expression analysis

Total RNA was extracted with TriReagent (Molecular Research Center, Inc., Cincinnati, OH) from cryopreserved mononuclear cell suspensions from patient bone marrow aspirates obtained at diagnosis. All gene expression microarrays were performed by the St. Jude Children’s Research Hospital, Hartwell Center for Bioinformatics & Biotechnology. High-quality RNA was hybridized to the HG-U133A (GPL96) or HGU133 Plus 2.0 (GPL570) oligonucleotide microarrays in accordance with the manufacturer’s protocol (Affymetrix, Santa Clara, CA). These microarrays contain 22,283 or 54,675 gene probe sets, representing approximately 18,400 or 47,400 human transcripts, respectively. Gene expression data were MAS5 [30] processed using the affy [31] Bioconductor [32] R-project package or using Affymetrix Microarray Suite version 5.0 [33,34] as previously described [35]. The gene expression data are available via http://trident.stjude.org and http://www.stjuderesearch.org/evans/.

### MicroRNA expression analysis

Total RNA was extracted with TriReagent (Molecular Research Center, Inc., Cincinnati, OH) from cryopreserved mononuclear cell suspensions from patient bone marrow aspirates obtained at diagnosis. All microRNA expression microarrays were performed by the St. Jude Children’s Research Hospital, Hartwell Center for Bioinformatics & Biotechnology. High-quality RNA was hybridized to miRCURY LNA 10.0 generated from ready to spot probe sets or preprinted 5th generation miRCURY LNA microRNA microarrays in accordance with the manufacturer’s protocol (Exiqon, Woburn, MA). Background subtracted minimum translated data were log2 transformed and then quantile normalized prior to statistical analysis. The microRNA expression data are available for download via http://trident.stjude.org, and http://www.stjuderesearch.org/evans/.

### Computational binding methods

First order free energy calculations were performed on each possible base pair permutation in a constrained microRNA-DNA triplex and these pair-wise interaction energies are then summed for each base pair in the triplex. Derived binding energies are listing in the supplemental material.

Restricted geometry optimizations were done via Gaussian03 using B3LYP/6-31g(*p*,*d*) to obtain the interaction energies of the base pairs. The model systems were constrained such that the nucleic acid ring systems remained co-planar to account for the steric hindrance that would be present in the experimental environment. Solvent effects were modeled using the PCM method [36] with water as the solvent. Differences between the isolated RNA and DNA components were taken to determine the interaction energies on a pairwise basis.

### Surface plasmon resonance

Duplex DNA and RNA were manufactured by Integrated DNA Technologies (Coralville, Iowa). Duplex DNA strand 1 (sense): 5’-CTGCTAGCTACTGGGGGAAGAAGAGGGGGCAGAGCTGCTAGCTACT-3’; strand 2 (antisense): 5’-AGTAGCTAGCAGCTCTGCCCCCTCTTCTTCCCCCAGTAGCTAGCAG-3’; synthesized hsa-miR-483-5p: 5’-AAGACGGGAGGAAAGAAGGGAG-3’. SPR experiments were conducted at 25°C using a Biacore 3000 optical biosensor (GE Healthcare). Streptavidin (Thermo Scientific) was covalently immobilized on a polycarboxylate hydrogel-coated gold surface (HC200m chip; Xantec Bioanalytics) using routine amine coupling chemistry in immobilization buffer (10 mM HEPES pH 7.4, 150 mM NaCl, 0.005% Tween20). Carboxyl groups on the hydrogel were activated with N-ethyl-N’-(3-dimethylaminopropyl) carbodiimide (EDC) and N-hydroxysuccinimide (NHS), and streptavidin was injected in 10 mM sodium acetate pH 4.5 until immobilization levels of 6000 RU were achieved. Remaining active sites were blocked by reaction with ethanolamine.

Nucleic acid oligomers were dissolved in TE buffer (10 mM Tris pH 8.0, 1 mM EDTA) and diluted in binding buffer (10 mM Tris pH 8.0, 100 mM NaCl, 10 mM MgCl_2_, 0.02% Tween20) before injection over the chip. Biotinylated single stranded RNAs were injected over the streptavidin surfaces until ~30 RU were captured. For manual test injections, data are shown as single-referenced sensorgrams of 20-fold dilutions of the DNA stocks (final concentrations ~2–5 μM) injected over the RNA surfaces. For the kinetic analysis, duplex DNA was prepared as a 2-fold dilution series starting at 20 nM and was injected in triplicate at each concentration at a flow rate of 75 μL/min. The chip was regenerated between cycles with a 20 second injection of 1 mM NaOH + 1 M NaCl. The data were processed, double-referenced and globally fit to a 1:1 binding model [37] using the software package Scrubber2 (version 2.0c, BioLogic Software). The equilibrium affinity constant (*K*_D_) was calculated as the quotient of the kinetic rate constants (*k*_d_/*k*_a_).

### Fluorescence resonance energy transfer

Duplex DNA and RNA were manufactured by Integrated DNA Technologies (Coralville, Iowa). Duplex DNA strand 1 (sense): 5’-CTGCTAGCTACTGGGGGAAGAAGAGGGGGCAGAGCTGCTAGCTACT-3’; strand 2 (antisense): 5’-AGTAGCTAGCAGCTCTGCCCCCTCTTCTTCCCCCAGTAGCTAGCAG-3’; synthesized hsa-miR-483-5p with a 3’ ROX label: 5’-AAGACGGGAGGAAAGAAGGGAG-ROX-3’. SYBR Green II (Life Technologies, Grand Island, NY) was used as the intercalating dye for duplex DNA. Reaction mixes were plated into 384 well black flat bottom plates and read using a Synergy H4 Hybrid Reader (Biotek, Winooski, VT) withGen5 software. SYBR Green II is excited, and an increase in ROX emission is measured to detect binding. Reactions were carried out at physiological pH and temperature.

### Electrophoretic Mobility Shift Assay (EMSA)

DNA and RNA oligos were manufactured by Integrated DNA Technologies (Coralville, Iowa). A stock solution of 10 μM ROX-labeled hairpin duplex DNA (ROX-5’-TGGGGGAAGAAGAGGGGGCAGAGATTTTTCTCTGCCCCCTCTTCTTCCCCCA-3’) was prepared in 10 mM Tris pH 7.4, heated at 95°C for 5 minutes to fully denature, followed by annealing of the 24-nucleotide sense and antisense regions (cooling to 22°C at a rate of 0.1°C/sec). Stock solutions of 200–1000 μM triplex forming oligos (TFOs) were prepared in nuclease-free distilled water. The following RNA and DNA TFOs were tested for binding to the duplex DNA: hsa-mIR-483-p (5’-AAGACGGGAGGAAAGAAGGGAG-3’ with 16 favorable Hoogsteen bonds), Hoogsteen bond-optimized hsa-mIR-483-p (483-opti, 5’-GAGACGGGGGAGAAGAAGGGGG-3’ with 21 favorable Hoogsteen hydrogen bonds), scrambled microRNA (483-scramble, 5’-GGAAGGGCAGGGAGGGGGAAGA-3’ with 10 favorable Hoogsteen bonds), truncated hsa-mIR-483-p (L-11nt-opti, 5’-GAAGAAGGGGG-3’ and R-11nt-opti, 5’-GAGACGGGGGA-3’, with 11 and 10 favorable Hoogsteen bonds, respectively). Binding reactions contained 0.1 μM ROX-labeled hairpin duplex DNA, in presence or absence of 5 μM DNA or RNA TFO in 1x binding buffer (10 mM Tris pH 7.4, 125 mM NaCl, 6 mM MgCl_2_, 0.1 mM Spermine) in a volume of 10 μl; incubated at 22°C for 3 hrs. In competition assays between DNA-TFO and RNA-TFO for binding to duplex DNA, to mixtures of 0.1 μM ROX-labeled hairpin duplex DNA and 5 μM 483-opti DNA-TFO, increasing amounts (30, 60, 150 μM) of RNA-TFOs were added, and incubated as mentioned above. Reactions were supplemented with 2 μl 6x Gel Loading Solution Type I (Sigma-Aldrich, Saint Louis, Missouri) and analyzed by electrophoresis at 50 V on 20% native acrylamide mini gels (19:1 acrylamide/bisacrylamide) in 1xTBE, 125 mM NaCl, 8 mM MgCl_2_; at 4°C for 16–24 hrs. After electrophoresis the gels were imaged on an Odyssey imager at 600 nm, and duplex and triplex signals were quantified using Image Studio Software (Li-Cor Biosciences-Biotechnology, Lincoln, Nebraska).

### Identification of microRNA, genomic DNA binding sites

An algorithm (‘Trident’) to identify microRNA, genomic DNA binding sites was developed in C and several post-processing pipelines were created (see Supplement). Extending the techniques developed by Betel et al. [38], the Trident algorithm takes known microRNA transcripts and searches genomic DNA for potential binding sites. MicroRNA sequences were obtained from mirbase http://www.mirbase.org/ version 19. Genomic DNA sequences for fifteen species (shown in Fig 1) were obtained from National Center for Biotechnology Information, U.S. National Library of Medicine (NCBI/NLM) via anonymous file transfer protocol (FTP).

Trident performs a search of microRNA—DNA triplex forming sites by assigning both a heuristic score and base pair binding energy to each possible alignment of microRNA and DNA strands. For each alignment location, Trident calculates energy and score values for Direct and Indirect Hoogsteen and Reverse Hoogsteen binding types. If heuristic score and energy exceed specified thresholds, the matching site is reported. Memory usage is directly proportional to the DNA sequence length. Therefore, genome sequences were segmented so that the binding site search could be run in parallel on a compute cluster and an in-house distributed grid [39]. Overlap between each segment is provided to account for the boundary between two neighboring segments. After the binding site search has finished, post-processing is performed on all Trident results to classify relative fitness of matches intra-genomically.

To demonstrate relative fitness, heuristic score and energy pairs were classified within each genome based on their relative values. Frequencies for each energy-score pair were analyzed and ranked by percentile, which was used to classify ranks into five match classifications. Linear interpolation was then used to classify arbitrary energy-score pairs. Random DNA sequences were generated by stochastically selecting A, T, G, or C. Although dinucleotide content across genomes may be heterogeneous [40], we did not adjust the nucleotide frequency for each individual genome, rather used a non-biased frequency of 0.25 for each nucleotide for all analyzed genomes.

### Molecular Dynamics (MD) simulations

All simulations were performed by AMBER12 with force field ff10 and generalized Born (GB) model. The reverse sequence of the selected microRNA, which has less potential to form favorable reverse Hoogsteen pairs, was also constructed as a negative control. The initial conformation of B form DNA duplex and microRNA were generated by 3DNA. The starting complex structures were constructed by simulated annealing with positional restraints of DNA duplex and NMR distance restraints of Hoogsteen hydrogen bonds. The positional restraints of DNA duplex were then removed and a 10ns MD simulation was performed on each system with Watson-Crick pair and reverse Hoogsteen pair restraints. This was followed by a final 10ns MD production run which was performed after gradually removing all the distance restraints in 3ns for each system.

### NMR spectroscopy

Lyophilized RNA (hsa-miR-483-5p, 5’-AAGACGGGAGGAAAGAAGGGAG-3’), DNA with the same sequence, and hairpin duplex DNA (5’-TGGGGGAAGAAGAGGGGGCAGAGATTTTTCTCTGCCCCCTCTTCTTCCCCCA-3’) were purchased from Integrated DNA Technologies (Coralville, Iowa) and Life Technologies (Carlsbad, California). Hairpin duplex DNA was prepared by heating the duplex DNA oligo at 95°C for 5 minutes to fully denature, followed by annealing of the 24-nucleotide sense and antisense regions (cooling to 22°C at a rate of 0.1°C/sec). DNA and RNA oligos were either HPLC-purified or dialyzed. The nucleic acid sample concentration was 250 μM in 15 mM sodium phosphate, 150 mM KCl In 0.5 ml of 90% H_2_O and 10% D_2_O (pH = 7.5). NMR experiments were measured on a Bruker 600 MHz spectrometer equipped with a ^1^H and ^13^C detect, TCI triple resonance cryogenic probe using standard Bruker pulse programs. 2D [^1^H, ^1^H] TOCSY (Total Correlation Spectroscopy) spectra were acquired with 2048 X 256 points with 80 transients per increment with 70 ms mixing time at 298 K on free DNA duplex and in complex with RNA (1:1.5) and DNA (1:1) of the same sequence. All the spectra were processed using Topspin 3.2 and were analyzed in CARA [41].

## Data access

All data are available for download and browsing via http://trident.stjude.org and http://www.stjuderesearch.org/evans/.

## Supporting Information

S1 Fig MicroRNAs with mixed purine and pyrimidine content do not form triplex structures. Labeled microRNAs (3’ biotin) with mixed purine and pyrimdine content (hsa-miR-98: 5’-UGAGGUAGUAAGUUGUAUUGUU-3’ and hsa-miR-1: 5’-UGGAAUGUAAAGAAGUAUGUAU-3’), were immobilized and duplex DNA (Duplex A: Strand 1: 5'-TCATCGATCGTCAAAGAAAGAAGAAAAGAAAGGATCATCGATCGTC-3', Strand 2: 5’-GACGATCGATGATCCTTTCTTTTCTTCTTTCTTTGACGATCGATGA-3’, Duplex B: Strand 1: 5’-TCATCGATCGTCAAGAAAAGAAGAAAGAAGGAGATCATCGATCGTC-3’, Strand 2: 5’-GACGATCGATGATCTCCTTCTTTCTTCTTTTCTTGACGATCGATGA-3’) was introduced via injection. No detectable binding was observed for any combination of hsa-miR-1 (**B** and **D**) or hsa-miR-98 (panels **A** and **C**) with either Duplex A (**A** and **B**) or Duplex B (**C** and **D**).
(TIF)

S2 Fig **Short polypurine RNAs do not form triplexes with double stranded DNA (A) EMSA.** 5’ ROX-labeled hairpin duplex DNA (0.1 μM) was incubated for 3-hrs at 22°C in the presence (lanes 2–8) or absence (lane 1) of 2.5 μM L-11nt-DNA (5’-GAAGAAGGGGG-3’), and increasing concentration (30, 60, 150 μM) of competing 11-nucleotide Hoogsteen bond-optimized truncated hsa-miR-483-5p (L-11nt-RNA, 5’-GAAGAAGGGGG-3’, lanes 3–5; or R-11nt-RNA, 5’-GAGACGGGGGA-3’, lanes 6–8). Nucleic acids were resolved on a 20% non-denaturing acrylamide gel, and the ROX-signal visualized. In all 8 lanes only duplex DNA is detected; no triplex consisting of duplex DNA and either L-11nt-RNA, R-11nt-RNA or L-11nt-DNA is detected in any of the lanes. This indicates that, unlike the 22-nucleotide polypurine RNA strand (Fig 4), short polypurine (11-nucleotide) RNAs are incapable of triplex formation with duplex DNA. (**B, C**) **NMR**; Two-Dimensional (2D) [^1^H, ^1^H] TOCSY spectra of free double stranded DNA (red contours), and double strand DNA combined with an 11-nucleotide truncated hsa-miR-483-5p RNA oligo (5’-AAAGAAGGGAG-3’; purple contours; 1:1 complex) for thymidine nucleotides cross-peaks between H6 and H7 (**A**; methyl) and cytosine cross-peaks between H5 and H6 (**B**). For both thymidine and cytosine, the two spectra completely overlap with each other, indicating that, unlike the 22-nucleotide full length hsa-miR-483-5p (Fig 5), a short (11-nucleotide) polypurine RNA strand does not bind to double stranded DNA.
(TIF)

S3 Fig Genome-wide predictions of microRNA binding to duplex DNA in multiple species. The interactive data dashboard (http://trident.stjude.org) provides downloads of genome-wide predictions of microRNA, double stranded DNA binding in 15 different species. In some instances for genomes with wider interest across the scientific community (*Caenorhabditis elegans*, *Danio rerio*, *Homo sapiens*, *Mus musculus*, *and Rattus norvegicus*) links to a genome browser are available to view Grade 1 Trident binding sites in their genomic context.
(TIF)

S4 Fig Trident triplex prediction tool. To facilitate usage of the Trident algorithm the interactive data dashboard (http://trident.stjude.org) provides a web based prediction software implementation of the Trident. Users only need to provide their microRNA sequence and one strand of their duplex DNA sequence.
(TIF)

S5 Fig Trident triplex return. Upon submission of microRNA sequences, detailed results are returned by Tridentwith orwithout the option of interpolating the Trident grade based on available calculated species.
(TIF)

S6 Fig Trident search tool. Pre-calculated results from genomes with wide interest across the scientific community (*Caenorhabditis elegans*, *Danio rerio*, *Homo sapiens*, *Mus musculus*, *and Rattus norvegicus*) are available for searching at the interactive data dashboard (http://trident.stjude.org). Users only need to input a gene symbol to see which triplex binding sites are near that gene (Trident grades 1–4). Genomic Start, genomic end, score, energy and the binding motif are returned.
(TIF)

S7 Fig Trident source code. Source code for the trident executable is freely available under a GPLv3 license and compiles on Linux based, Mac OS X based and Windows based machine. Download instructions are available at (http://trident.stjude.org).
(TIF)

S8 Fig Hoogsteen and reverse Hoogsteen binding motifs. Double-helical DNA is capable of forming triple-helical structures through Hoogsteen and reverse Hoogsteen interactions in the major groove of the duplex, and we show physical evidence that microRNAs form triple-helical structures with duplex DNA, and identity microRNA sequences that favor triplex formation. We developed an algorithm (Trident) to search genome-wide for potential triplex-forming sites and show that several mammalian and non-mammalian genomes are enriched for strong microRNA triplex binding sites.
(TIF)

S9 Fig Schematic representation of Hoogsteen and reverse Hoogsteen binding. The major grove of DNA is capable of allowing a third oligonucleotide strand to interact. A schematic artistic representation of this shows the duplex DNA (yellow), interacting with a microRNA (blue).
(TIF)

S1 Algorithm Trident algorithm. Complete Trident Algorithm, lines 1 through 32.
(PDF)

S1 Text Supplemental Text. 1) Calculated thermodynamic energies of Hoogsteen and reverse Hoogsteen bonds. 2) Trident Download options. 3) Trident Postprocessing Summary.
(DOCX)
