# Supplementary figures and images for: MicroRNAs Form Triplexes with Double Stranded DNA at Sequence-Specific Binding Sites; a Eukaryotic Mechanism via which microRNAs Could Directly Alter Gene Expression
Source: PLoS Comput Biol. 2016 Feb 4;12(2):e1004744. doi: 10.1371/journal.pcbi.1004744 (PMC4742280; doi:10.1371/journal.pcbi.1004744)

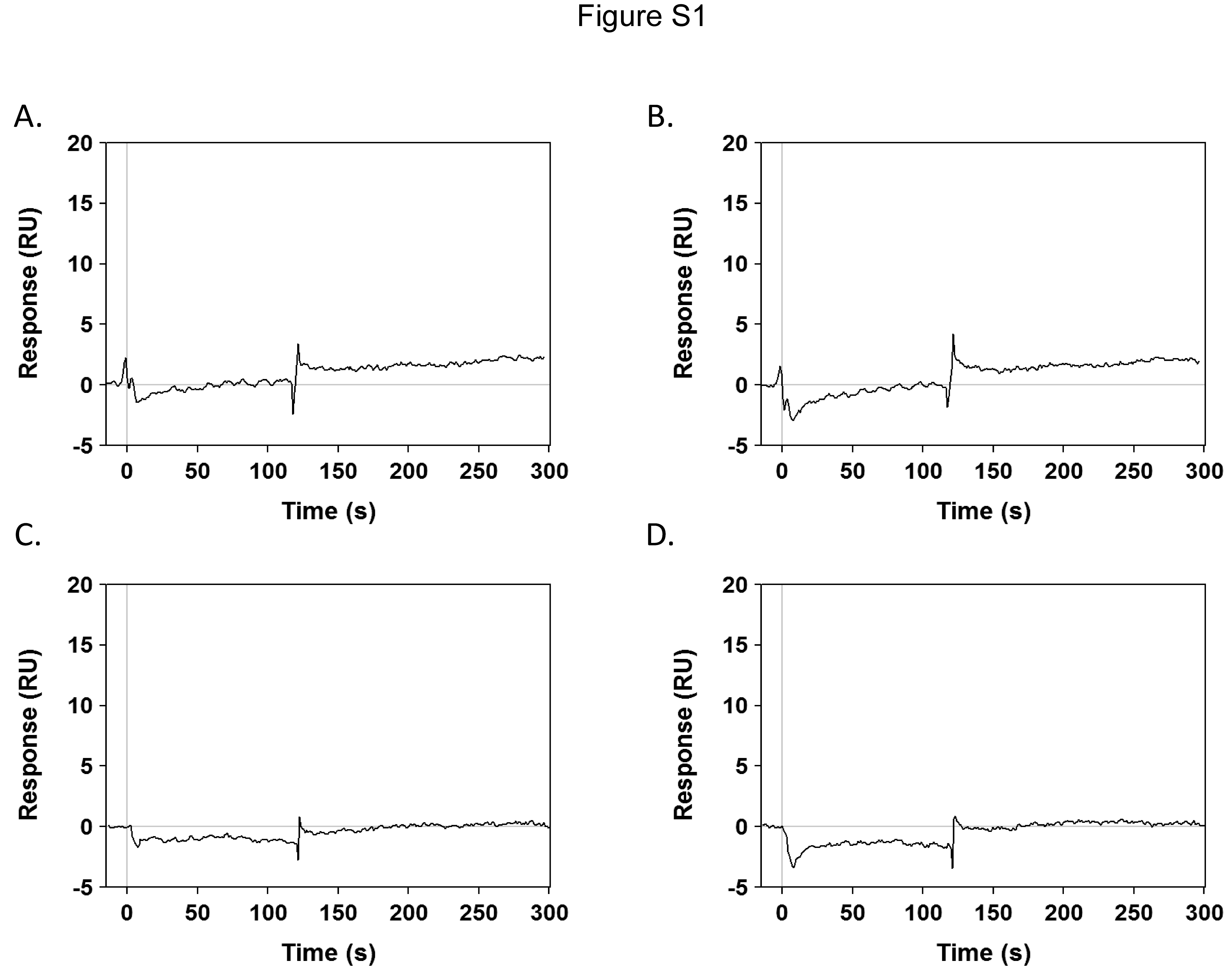

Supplement: S1 Fig — Labeled microRNAs (3’ biotin) with mixed purine and pyrimdine content (hsa-miR-98: 5’-UGAGGUAGUAAGUUGUAUUGUU-3’ and hsa-miR-1: 5’-UGGAAUGUAAAGAAGUAUGUAU-3’), were immobilized and duplex DNA (Duplex A: Strand 1: 5'-TCATCGATCGTCAAAGAAAGAAGAAAAGAAAGGATCATCGATCGTC-3', Strand 2: 5’-GACGATCGATGATCCTTTCTTTTCTTCTTTCTTTGACGATCGATGA-3’, Duplex B: Strand 1: 5’-TCATCGATCGTCAAGAAAAGAAGAAAGAAGGAGATCATCGATCGTC-3’, Strand 2: 5’-GACGATCGATGATCTCCTTCTTTCTTCTTTTCTTGACGATCGATGA-3’) was introduced via injection. No detectable binding was observed for any combination of hsa-miR-1 (B and D) or hsa-miR-98 (panels A and C) with either Duplex A (A and B) or Duplex B (C and D). (TIF) [file pcbi.1004744.s001.tif]

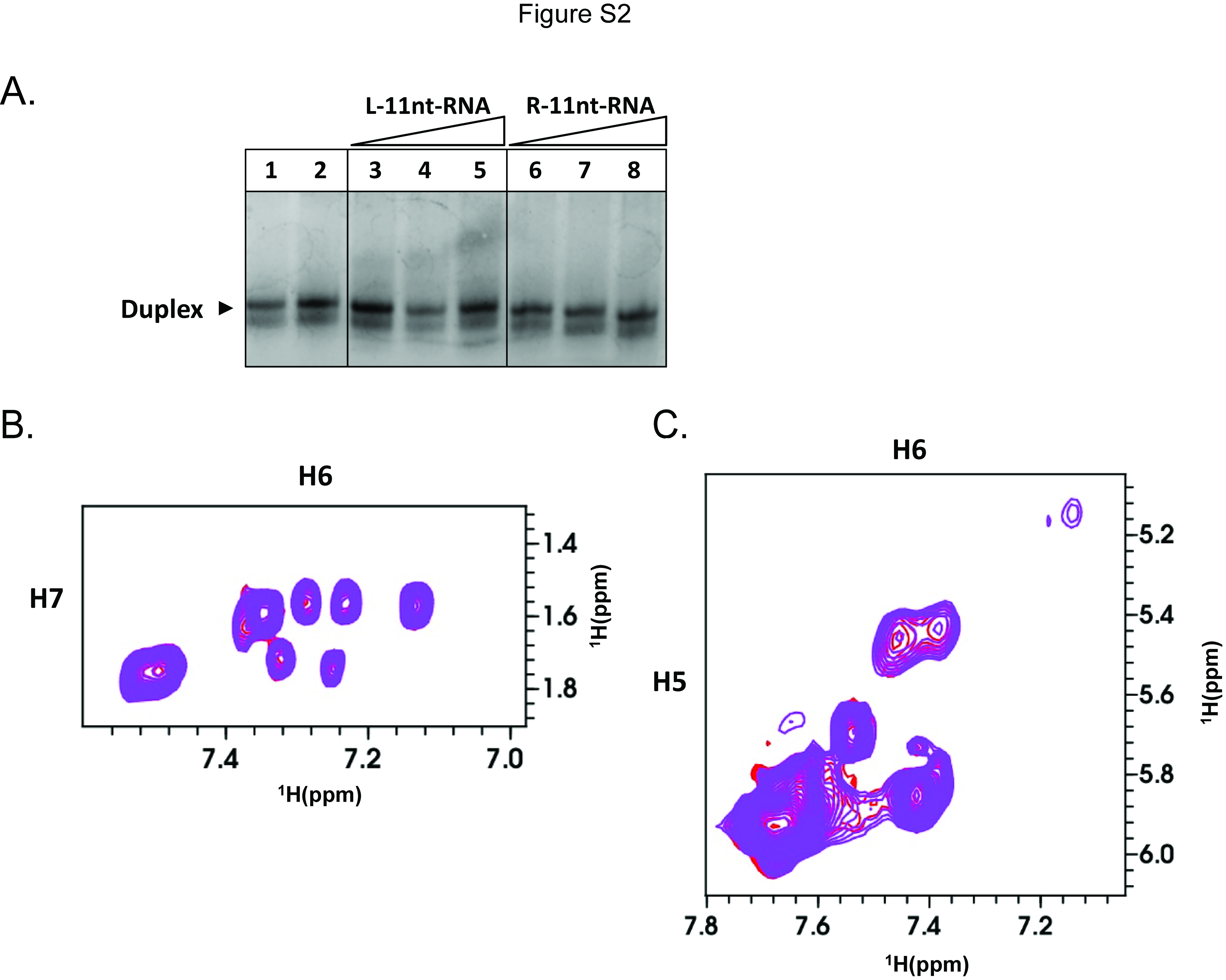

Supplement: S2 Fig — Short polypurine RNAs do not form triplexes with double stranded DNA (A) EMSA. 5’ ROX-labeled hairpin duplex DNA (0.1 μM) was incubated for 3-hrs at 22°C in the presence (lanes 2–8) or absence (lane 1) of 2.5 μM L-11nt-DNA (5’-GAAGAAGGGGG-3’), and increasing concentration (30, 60, 150 μM) of competing 11-nucleotide Hoogsteen bond-optimized truncated hsa-miR-483-5p (L-11nt-RNA, 5’-GAAGAAGGGGG-3’, lanes 3–5; or R-11nt-RNA, 5’-GAGACGGGGGA-3’, lanes 6–8). Nucleic acids were resolved on a 20% non-denaturing acrylamide gel, and the ROX-signal visualized. In all 8 lanes only duplex DNA is detected; no triplex consisting of duplex DNA and either L-11nt-RNA, R-11nt-RNA or L-11nt-DNA is detected in any of the lanes. This indicates that, unlike the 22-nucleotide polypurine RNA strand (Fig 4), short polypurine (11-nucleotide) RNAs are incapable of triplex formation with duplex DNA. (B, C) NMR; Two-Dimensional (2D) [1H, 1H] TOCSY spectra of free double stranded DNA (red contours), and double strand DNA combined with an 11-nucleotide truncated hsa-miR-483-5p RNA oligo (5’-AAAGAAGGGAG-3’; purple contours; 1:1 complex) for thymidine nucleotides cross-peaks between H6 and H7 (A; methyl) and cytosine cross-peaks between H5 and H6 (B). For both thymidine and cytosine, the two spectra completely overlap with each other, indicating that, unlike the 22-nucleotide full length hsa-miR-483-5p (Fig 5), a short (11-nucleotide) polypurine RNA strand does not bind to double stranded DNA. (TIF) [file pcbi.1004744.s002.tif]

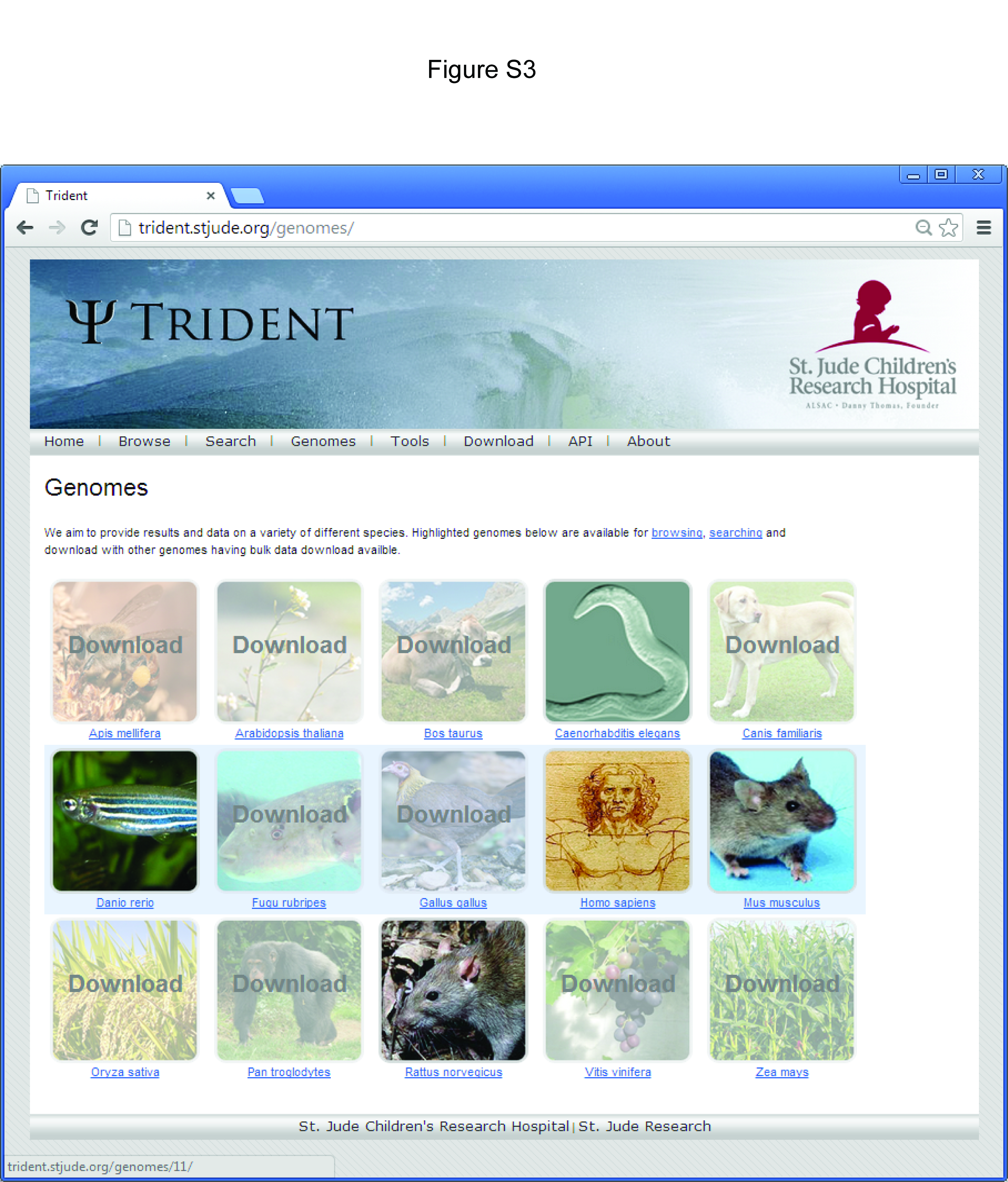

Supplement: S3 Fig — The interactive data dashboard (http://trident.stjude.org) provides downloads of genome-wide predictions of microRNA, double stranded DNA binding in 15 different species. In some instances for genomes with wider interest across the scientific community (Caenorhabditis elegans, Danio rerio, Homo sapiens, Mus musculus, and Rattus norvegicus) links to a genome browser are available to view Grade 1 Trident binding sites in their genomic context. (TIF) [file pcbi.1004744.s003.tif]

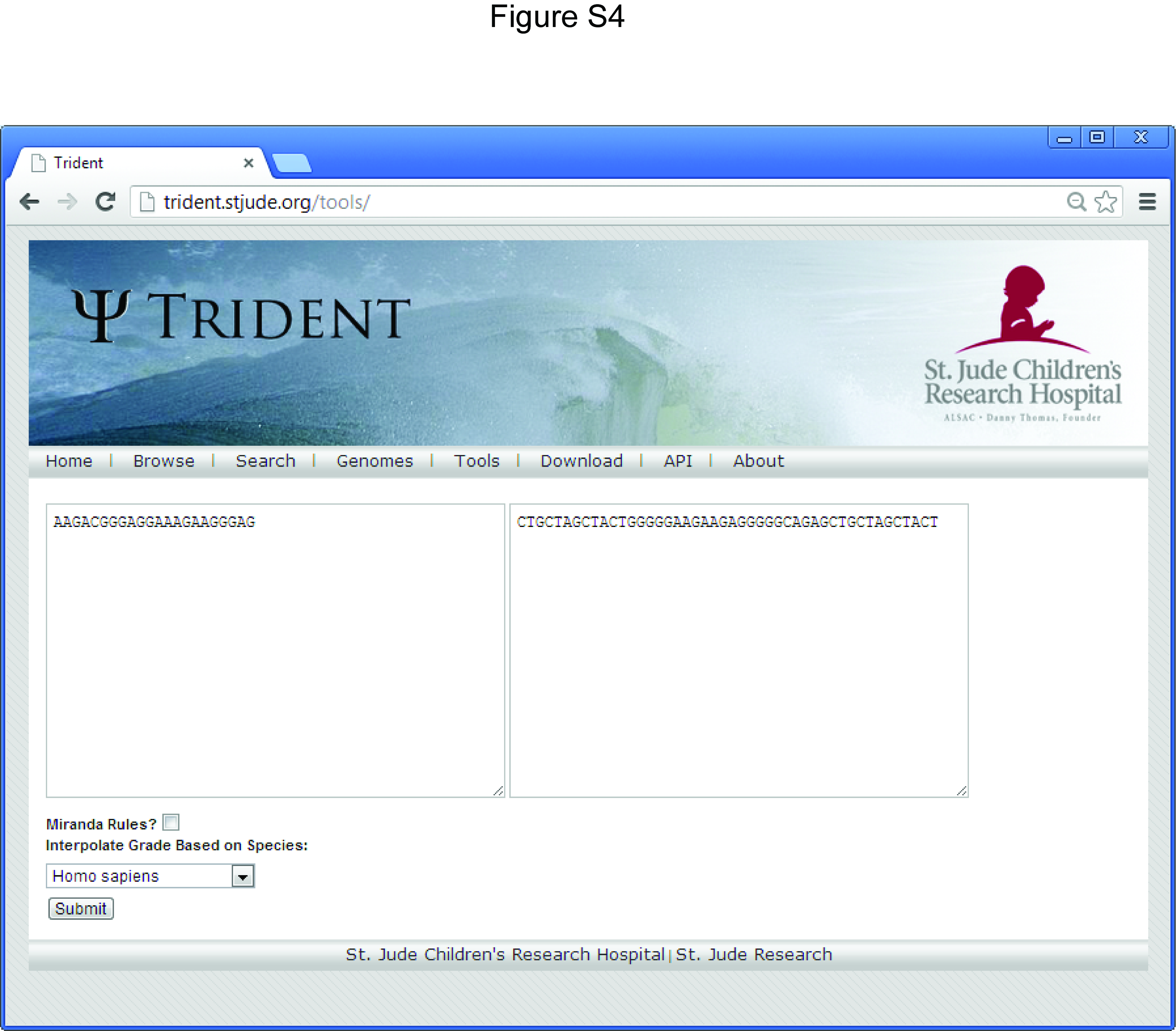

Supplement: S4 Fig — To facilitate usage of the Trident algorithm the interactive data dashboard (http://trident.stjude.org) provides a web based prediction software implementation of the Trident. Users only need to provide their microRNA sequence and one strand of their duplex DNA sequence. (TIF) [file pcbi.1004744.s004.tif]

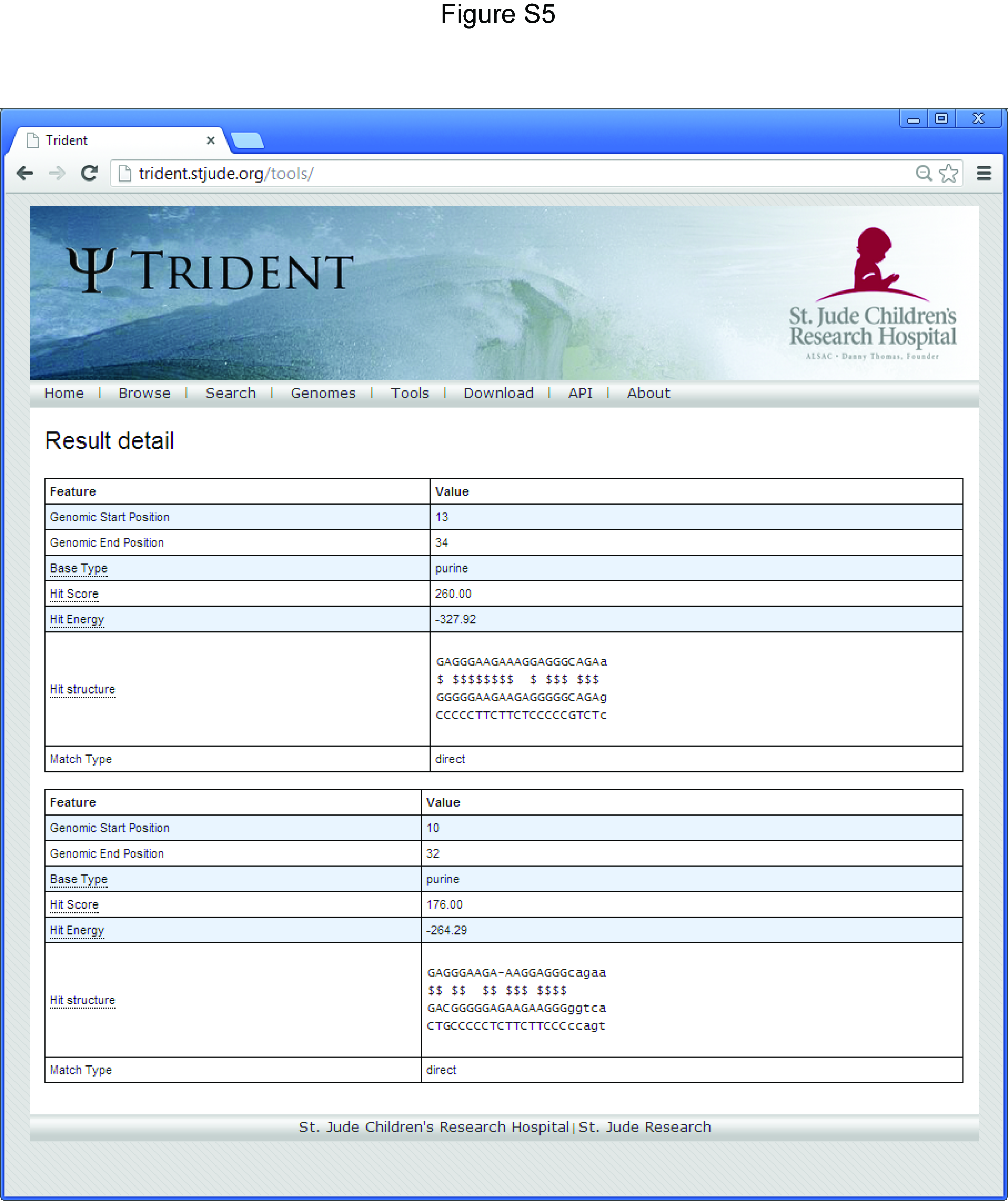

Supplement: S5 Fig — Upon submission of microRNA sequences, detailed results are returned by Tridentwith orwithout the option of interpolating the Trident grade based on available calculated species. (TIF) [file pcbi.1004744.s005.tif]

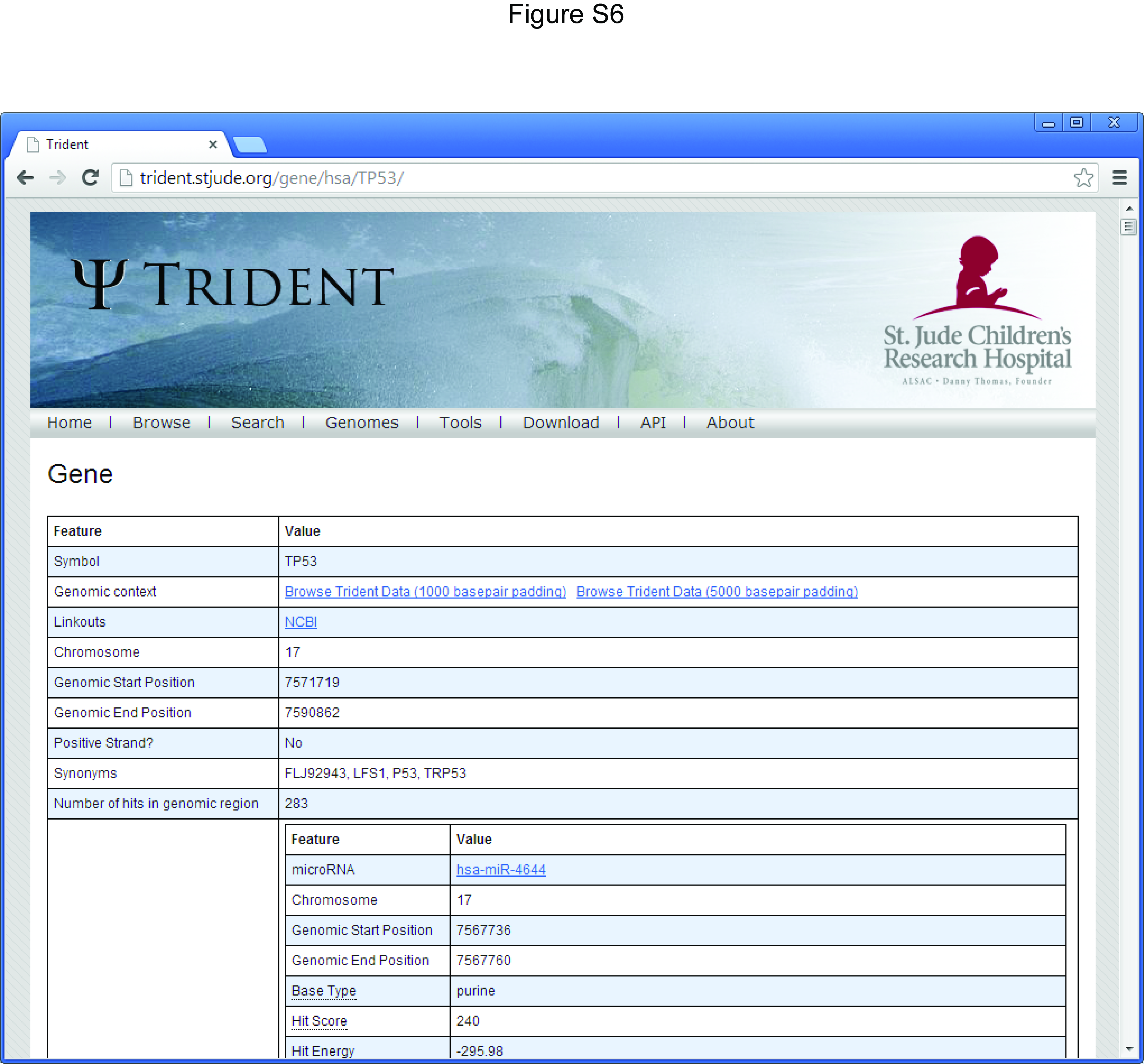

Supplement: S6 Fig — Pre-calculated results from genomes with wide interest across the scientific community (Caenorhabditis elegans, Danio rerio, Homo sapiens, Mus musculus, and Rattus norvegicus) are available for searching at the interactive data dashboard (http://trident.stjude.org). Users only need to input a gene symbol to see which triplex binding sites are near that gene (Trident grades 1–4). Genomic Start, genomic end, score, energy and the binding motif are returned. (TIF) [file pcbi.1004744.s006.tif]

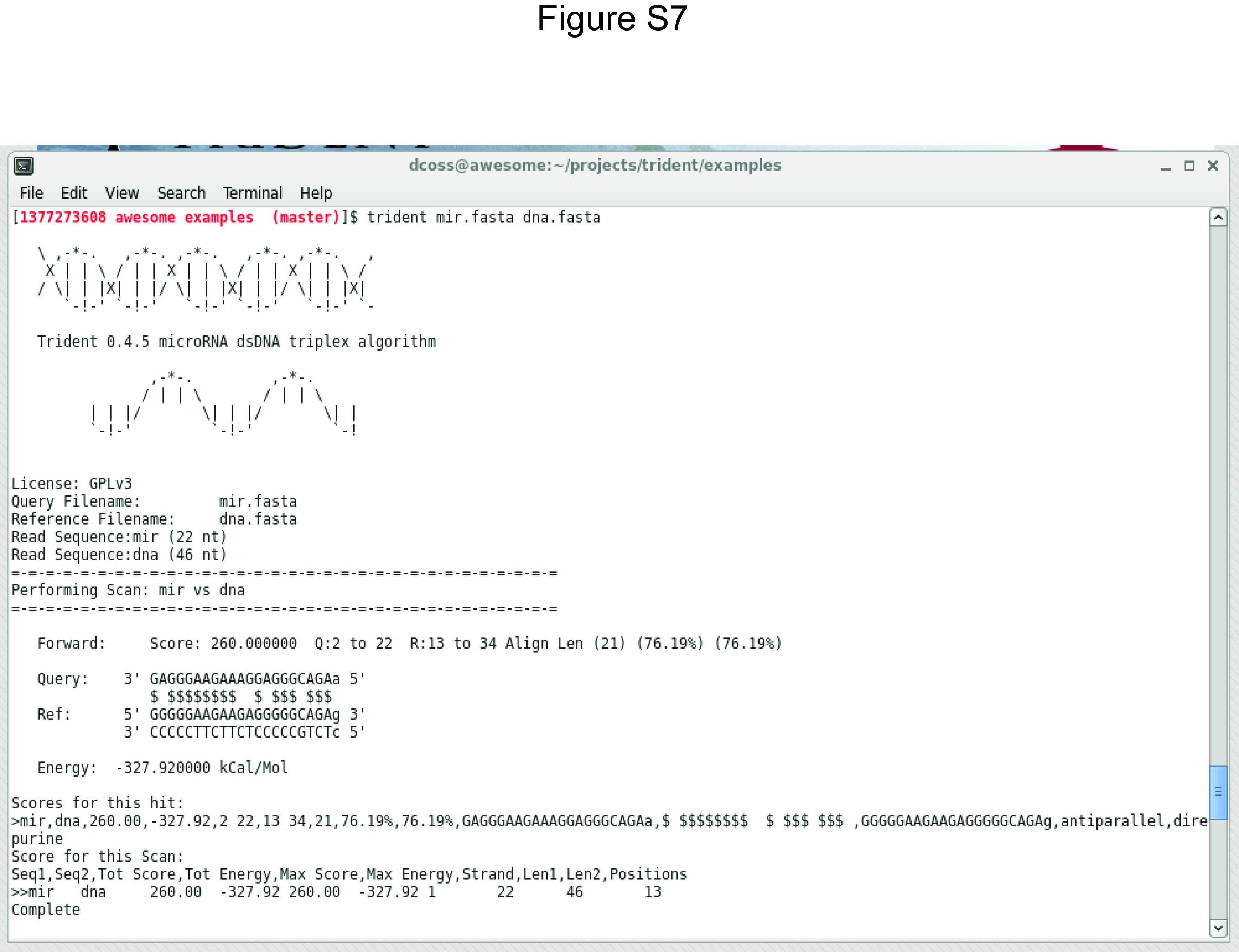

Supplement: S7 Fig — Source code for the trident executable is freely available under a GPLv3 license and compiles on Linux based, Mac OS X based and Windows based machine. Download instructions are available at (http://trident.stjude.org). (TIF) [file pcbi.1004744.s007.tif]

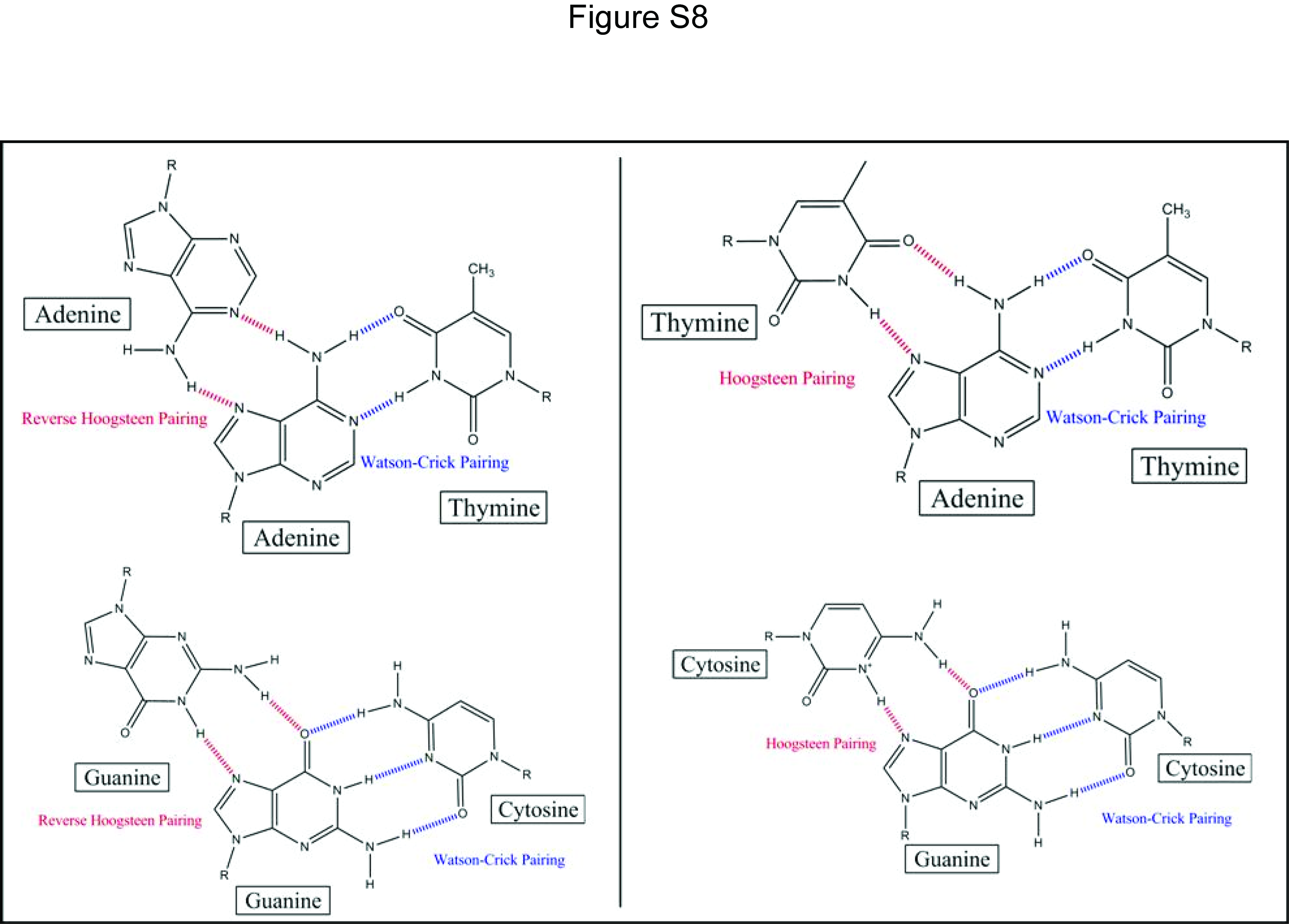

Supplement: S8 Fig — Double-helical DNA is capable of forming triple-helical structures through Hoogsteen and reverse Hoogsteen interactions in the major groove of the duplex, and we show physical evidence that microRNAs form triple-helical structures with duplex DNA, and identity microRNA sequences that favor triplex formation. We developed an algorithm (Trident) to search genome-wide for potential triplex-forming sites and show that several mammalian and non-mammalian genomes are enriched for strong microRNA triplex binding sites. (TIF) [file pcbi.1004744.s008.tif]

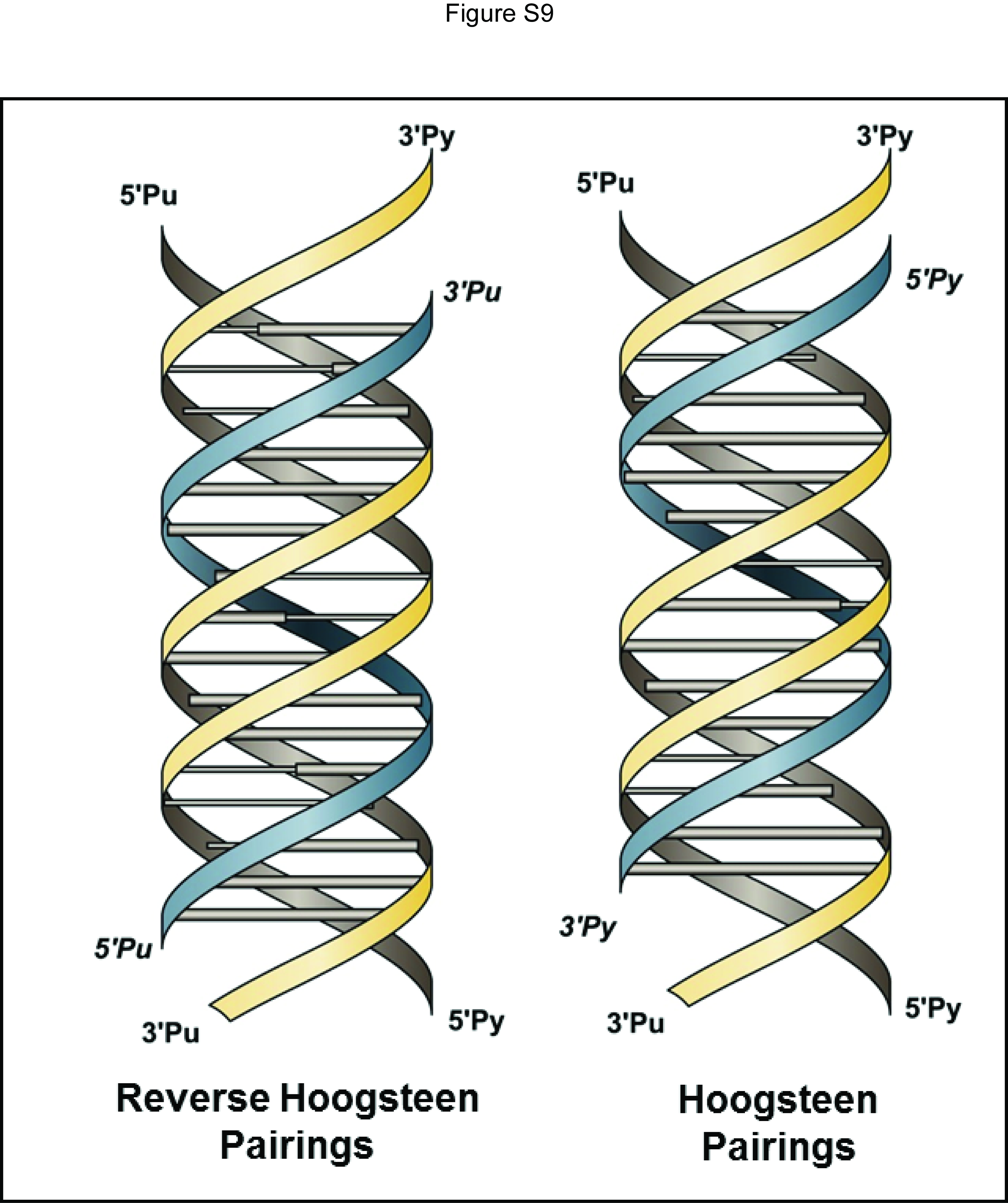

Supplement: S9 Fig — The major grove of DNA is capable of allowing a third oligonucleotide strand to interact. A schematic artistic representation of this shows the duplex DNA (yellow), interacting with a microRNA (blue). (TIF) [file pcbi.1004744.s009.tif]
