# Supplementary material for: MicroRNAs Form Triplexes with Double Stranded DNA at Sequence-Specific Binding Sites; a Eukaryotic Mechanism via which microRNAs Could Directly Alter Gene Expression
Source: PLoS Comput Biol. 2016 Feb 4;12(2):e1004744. doi: 10.1371/journal.pcbi.1004744 (PMC4742280; doi:10.1371/journal.pcbi.1004744)
Supplement: S1 Algorithm — Complete Trident Algorithm, lines 1 through 32. (PDF) [file pcbi.1004744.s010.pdf]

Trident Algorithm

Algorithm 1: Calculate microRNA, double strand DNA triplex forming potential

```
for all —query_sequence— do
    for all —reference_sequence— do
        for all —match_types— do
            run do_alignment
        end for
    end for
end for
```

Algorithm 2: do_alignment

```
for query_sequence i = 1..N do
    if i is in end region then
        open ← −9
        ext ← −4
    else
        open ← −36
        ext ← –16
    end if
    for reference_sequence j = 1..M do
        if weighted_region then
            nt_nt_score_{i,j} ←score5p(i, j)
        else
            nt_nt_score_{i,j} ← score(i, j)
        end if
        maxv(x, y, z) ≡ max(x, y, z, 0)
        A(i, j) ≡ score(i, j) + maxv(A(i −1, j −1))
        B(i, j) ≡ max(A(i, j −1) + open, B(i, j −1))
        C(i, j) ≡ { max(C(i −1, j) + ext, A(i −1, j) + open    if in weighted region
                  { −1                                         else
        best_{i,j} ← max(A(i, j),B(i, j),C(i, j))
        if best_{i,j} is within allowed score ranges then
            retain hit struct
        end if
    end for
end for
Remove overlapping hit structs
Sort hit structs by score, DNA position and MicroRNA position
for all Hit structs do
    for all Positions in MicroRNA - DNA alignment, i and j, respectively do
        if best_{i,j} = A(i, j) then
            Label base pairs as bonded
        else if best_{i,j} = B(i, j) then
            Mark microRNA, i, as a gap
```

```
        else
          Mark DNA, j, as a gap
        end if
    end for
end for
```
