# Supplementary material for: MicroRNAs Form Triplexes with Double Stranded DNA at Sequence-Specific Binding Sites; a Eukaryotic Mechanism via which microRNAs Could Directly Alter Gene Expression
Source: PLoS Comput Biol. 2016 Feb 4;12(2):e1004744. doi: 10.1371/journal.pcbi.1004744 (PMC4742280; doi:10.1371/journal.pcbi.1004744)
Supplement: S1 Text — 1) Calculated thermodynamic energies of Hoogsteen and reverse Hoogsteen bonds. 2) Trident Download options. 3) Trident Postprocessing Summary. (DOCX) [file pcbi.1004744.s011.docx]

**Supplemental Text**

1. **Calculated thermodynamic energies**

| **Pairing Type** | **Double strand**  **bases** | **Third strand**  **base** | **Triplex**  **representation** | **Energy**  **(kcal/mol)** |
| --- | --- | --- | --- | --- |
| Hoogsteen | TA | U | TA:U | -9.94 |
| Hoogsteen | CG | C | CG:C | -28.18 |
| Reverse Hoogsteen | TA | A | TA:A | -9.77 |
| Reverse Hoogsteen | CG | G | CG:G | -26.93 |

“open” and “ext” scoring values were taken from miRanda.

1. **Trident Download Options**

There are three main components to Trident. Initial results are produced with a single executable written in C. Additionally, there is a set of post-processing tools available to

analyze trident data. Finally, data may be presented using the web user interface described below. All three are available under the GNU General Public License version 3. These components may be obtained individually (below) or together in a pre-built Linux system. Trident was built and tested on CentOS 6. However, the trident executable has been used on Mac OSX 1.6 and Windows XP and 7, with the Windows version being built using Cygwin.

**Trident Executable**

*Obtaining Trident*

The Trident executable is available as source code or a prebuilt linux package. The current release may be downloaded at http://trident.stjude.org. Also, the source code repository is available using git. A copy of the source code can be obtained by at the Github St. Jude repository: github.com/stjude.

*Building Trident*

Trident is written in C and is built using GNU Autotools. To build trident, in a terminal, change to the top Trident source directory. Then run

./configure && make

**Trident Hadoop**

Depending on the score threshold, trident output can be very lengthy. To facilitate efficient and simple data extraction and analysis, the trident package comes with Hadoop Map Reduce programs. The latest trident score map reduce program is available as a jar file. Also, the hadoop code is located in the hadoop subdirectory of the main source code distribution. The java main class is TridentScoreMR. This program requires an input file name as a command line argument. For example, to count the number of Energy-Score pairs, run:

hadoop jar TridentScoreMR.jar TridentScoreMR /user/dcoss/hs/chr18/chr18.out

**Trident Python**

Multiple python scripts are available for preparing and analyzing data from trident. For a detailed description of the trident modules available, see the trident python api page (http://trident.stjude.org/api). The python code is located in the python subdirectory of the main source code distribution. Also, for linux, the trident python package and scripts may be downloaded as a Python Egg at http://trident.stjude.org/download/.

The trident python code may be built from source using setuptools. To build and install

the python code, in the python subdirectory, run:

python setup.py install

**Trident Web**

A web framework was developed to allow the user to search and visualize trident results. A template trident website is available as a django application. The source may downloaded as a source package or cloned from the git repository at github.com/stjude

Additionally, the trident linux system contains a working copy of trident web.

The website requires Apache, Python 2.7 and Django 1.5. To setup Apache to run trident web, the apache configuration files must be changed to include the trident web path. An example apache configuration file is located in the apache subdirectory of the TridentWeb directory.

1. **Trident Postprocessing Summary**

• Count Energy-Score and microRNA frequencies (Hadoop)

• Combine frequencies (Python)

• Rank energy/score pairs (Python)

• Generate Energy and Score distributions (Python)

• Generate plots (Python)

• Interpolate Energy-Score grades from energy/score rankings (Python)
